# Identification of the Key Genes Involved in the Tumorigenesis and Prognosis of Prostate Cancer

**DOI:** 10.1155/2022/5500416

**Published:** 2022-10-05

**Authors:** Wenxuan Wang, Qinghui Wu, Ali Mohyeddin, Yousheng Liu, Zhitao Liu, Jianqiang Ge, Bao Zhang, Gan Shi, Weifu Wang, Dinglan Wu, Fei Wang

**Affiliations:** ^1^Department of Urology, Hainan General Hospital, Hainan Affiliated Hospital of Hainan Medical University, Haikou 570100, China; ^2^Department of Urology, Guangdong Hospital of Traditional Chinese Medicine, Zhuhai, Guangdong Province, 519015, China; ^3^Department of Plastic Surgery, Zhujiang Hospital, Southern Medical University, Guangzhou, Guangdong Province, 510280, China; ^4^Shenzhen Key Laboratory of Viral Oncology, The Clinical Innovation & Research Centre, Shenzhen Hospital, Southern Medical University, Shenzhen 518100, China

## Abstract

**Background:**

Prostate cancer (PCa) is a malignant tumor in males, with a majority of the cases advancing to metastatic castration resistance. Metastasis is the leading cause of mortality in PCa. The traditional early detection and prediction approaches cannot differentiate between the different stages of PCa. Therefore, new biomarkers are necessary for early detection and clear differentiation of PCa stages to provide precise therapeutic intervention.

**Methods:**

The objective of the study was to find significant differences in genes and combine the three GEO datasets with TCGA-PRAD datasets (DEG). Weighted gene coexpression network analysis (WGCNA) determined the gene set and PCa clinical feature correlation module utilizing the TGGA-PRAD clinical feature data. The correlation module genes were rescreened using the biological information analysis tools, with the three hub genes (TOP2A, NCAPG, and BUB1B) for proper verification. Finally, internal (TCGA) and external (GSE32571, GSE70770) validation datasets were used to validate and predict the value of last hub genes.

**Results:**

The hub gene was abnormally upregulated in PCa samples during verification. The expression of each gene was favorably connected with the Gleason score and TN tumor grade in clinical samples but negatively correlated with the overall survival rate. The expression of these genes was linked to CD8 naive cells and macrophages, among other cells. Antitumor immune cells like NK and NKT were favorably and adversely correlated with infiltrating cells, respectively. Simultaneously, the GSCV and GSEA indicated that the hub gene is connected with cell proliferation, death, and androgen receptor, among other signaling pathways. Therefore, these genes could influence the incidence and progression of PCa by participating in or modulating various signaling pathways. Furthermore, using the online tool of CMap, we examined the individual medications for Hughes and determined that tipifarnib could be useful for the clinical therapy of PCa.

**Conclusion:**

TOP2A, NCAPG, and BUB1B are important genes intimately linked to the clinical prognosis of PCa and can be employed as reliable biomarkers for early diagnosis and prognosis. Moreover, these genes can provide a theoretical basis for precision differentiation and treatment of PCa.

## 1. Introduction

PCa is one of the most frequent cancers in males and has the highest prevalence of male malignant tumors among the 112 nations in the global cancer statistics in 2020. Moreover, the fatality rate is second only to lung cancer patients [1].

The Gleason grading system [2] determines the aggressiveness of prostate cancer and judges the prognosis of patients [3, 4]. Prostate-specific antigen (PSA) and needle biopsy are the most common early screening methods. PSA can detect well-differentiated prostate cancer, although the difference between poorly differentiated and advanced prostate cancer is murky. In addition, PSA is also boosted by benign prostatic hyperplasia and prostatitis conditions. Thus, the occurrence of this gray zone creates a challenge in prostate cancer diagnosis.

The increase or decrease in prostate cancer mortality due to PSA screening does not accurately represent the survival rate of patients [5]. Furthermore, the Gleason score is subjective and inaccurate [6], and the biopsy scores of different pathologists could vary by 30-50% [7]. PSA and Gleason scores have not depicted correct differentiation or excellent predictive effects in these disorders. Therefore, finding early diagnostic and prognostic biomarkers, precisely distinguishing the various stages of PCa, and determining the exact therapy for PCa is critical.

The cumulative analysis of multiple data, numerous platforms, and substantial sample sizes has revealed certain advantages in screening various tumor markers due to the fast development and deployment of gene chips and second-generation sequencing technologies.

The current work utilized GEO and TCGA gene chip datasets to filter DEG and used the WGCNA, the Cytoscape software, and the cytoHubba plug-in the MCC technique to identify BUB1B, NCAPG, and TOP2A as hub genes. In addition, the HIPLOT online tool was used to study the potential biological functions of genes: the Gene Enrichment Analysis (GSEA), Gene Variation Analysis (GSVA), and Tumor Immune Infiltration Analysis (GSCA). The Cancer Genomics Database (cBioPortal) was used to study genetic changes of genes, and the Pharmacogenomics Database (CMAP) was used to screen prostate cancer-related small-molecule drugs.

## 2. Materials and Methods

### 2.1. PCa Gene Expression Dataset and Related Clinical Information

The GSE38241, GSE3325, and GSE46602 microarray datasets (Supplementary Table 1) were downloaded from the NCBI GEO dataset containing the tumor and normal prostate samples. In addition, the statistics and clinical information were downloaded from the TCGA database for TCGA-PRAD (RNA-seq) counts, including 498 tumor and 52 normal prostate samples.

### 2.2. Preliminary Screening of Candidate DEGs

Using the NCBI web analytical tool “GEO2R” (https://www.ncbi.nlm.nih.gov/geo/geo2r/), the differences between the selected GEO datasets were aggregated and evaluated to produce the differential gene expression data matrix (*P* ≤ 0.05 | LogFC | ≥1).

In addition, the “edgeR” (http://sangerbox.com/) transcriptome count data difference program evaluates differentially expressed genes between normal and tumor samples using the TCGA-PRAD RNA-seq counts data (TCGA_DEG *P* ≤ 0.05 | Log FC | ≥1).

The distinct genes from the four datasets were intersected and visualized using a Venn diagram.

### 2.3. WGCNA Analyzes Candidate DEGs and Identifies Vital Modules

The WGCN analysis established the relationships between distinct sample groups, gene modules, and genes having similar expression patterns.

The current analysis utilizes the TCGA-PRAD dataset. The WGCNA online analysis tool (http://sangerbox.com/) examined the candidate DEGs for hub genes associated with clinical feature-related modules. The soft threshold was set at 12 (scale-free *R*^2^ = 0.85), and the smallest module was set to three.

### 2.4. Hub Gene Protein Network Interaction

Using the Cystoscape v3.8.2 [8] software, a protein interaction network diagram was developed with the cytoHubba plug-in based on the best MCC by selecting the module having the highest association with clinical features (maximal clique centrality) [9]. The screening approach chose the top-ranked PPI hub gene while screening MMTC-hub gene for TC > 0.25 and MM > 0.4. Then, as the final hub gene, the overlapping parts of PPI hub gene and MMTC-hub gene were combined.

### 2.5. Hub Gene Clinical Characteristic Analysis

After screening the last hub genes, internal validation (TCGA dataset) and external validation (GSE70770 and GSE25371 dataset) were performed, respectively.

The HIPLOT online tool, named Between stats (https://hiplot.com.cn/), was used in conjunction with the TCGA-PRAD data to ascertain the differential expression of the hub genes between PCa and normal prostate tissue at varied Gleason scores and tumor TNM staging [10]. Analysis of variance (ANOVA) or Student's *t*-test was used to determine the statistical significance of the calculated findings.

Survival analysis, ROC curve drawing, and AUC calculation were conducted by The HIPLOT online tool to evaluate the diagnostic value of hub gene.

### 2.6. Prognostic Analysis of Hub Genes

The online tool for univariate survival analysis (http://sangerbox.com/) generated the KM survival curve, the ROC curve, and the area under the ROC curve (AUC) for evaluating the diagnostic value of the hub genes.

### 2.7. GSEA and GSVA

The 498 PCa samples in the TCGA-PRAD RNA-seq data were subdivided into the high-expression and low-expression groups based on the median expression of each hub gene, using the GSEA software [11] (GSEA3.0 https://www.gseamsigdb.org/gsea/index.jsp). The “c2.cp.kegg.v6.2.symbols.gmt” was used as the reference gene set (download from MSigDB [12]) for analysis. *P* ≤ 0.05 was considered statistically significant.

In addition, the internet database (http://bioinfo.life.hust.edu.cn/) was utilized in the RPPA (Reverse Protein Chip High-throughput Antibody Technology) [13], using the “GSVA” [14] R package. In the center, the pathways associated with PCa were scored, and the most related pathway to the hub gene was determined.

### 2.8. Analysis of Tumor-Infiltrating Immune Cells

The system examined the immune infiltration situation of the hub genes in PCa with the web tool GSCA [15] (http://bioinfo.life.hust.edu.cn/), and 550 samples in the TCGA PRAD data were retrieved. In addition, the expression of hub genes and 24 tumor-infiltrating immune cells (B cells, CD4 and CD8 T cells, NK cells, NKT cells, gamma delta T cells, neutrophils, macrophages, monocytes, and dendritic cells) were analyzed using the online tools.

### 2.9. Genetic Changes in the Hub Gene

The cancer genomics database cBioPortal (http://cbioportal.org) [16] utilized cancer genomics to study genetic changes related to hub genes.

### 2.10. Small-Molecule Drug Screening

CMAP online database (https://clue.io) analyzed the potential relationship between the hub genes and related drugs [17, 18].

DEGs of the green module were compared to CMap data to propose small-molecule therapies that could reverse the biological state of PCa. The standardized connection score of CMap ranged from –100 to +100. A positive link score meant the medicine could create signal biology in a specific disease, whereas a negative connection score indicated that the drug could prevent the signal biology. In general, the screening requirements for future study are points greater than or equal to +90 or points less than or equal to –90.

Touchstone and PCL screen genes were used from the same gene family or genes targeted by the same substance to find the best related small-molecule medications.

## 3. Results

### 3.1. Screening Candidate DEGs

**Figure 1:**
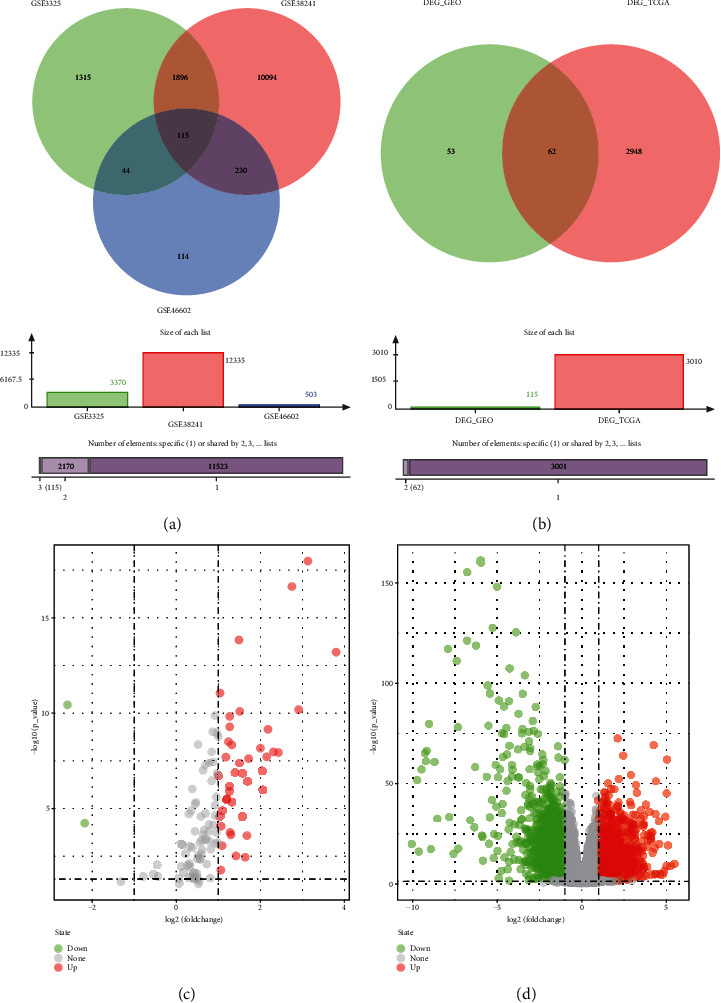
Screening candidate DEGs. (a) Number of the intersection from the GSE46602 (green circle), GSE38241 (red circle), and GSE3325 (blue circle) datasets. (b) Number of the intersection from the GSE-DEG (green circle) and the TCGA-PRAD (red circle) counts data. (c) Volcano plot of the integrated microarray of GSE46602, GSE38241, and GSE3325. The red nodes represent the upregulated DEGs, and the green node indicates downregulated DEGs. (d) Volcano plot of the integrated microarray of the top 100 TCGA-PRAD counts data. The red nodes represent upregulated DEGs, and the green node indicates downregulated DEGs.

We obtained 115 DEG (*P* ≤ 0.05 | LogFC | ≥1) from the intersection between GSE46602, GSE3325, and GSE38241 datasets (Figure 1(a)), including 108 upregulated and seven downregulated genes, to assess the DEG between PCA and normal tissues (Figure 1(c), Supplementary Figure 1.1). Supplementary Table 2 shows the details of the three DGE datasets.

The DEG of the TCGA_PRAD dataset yielded 3010 DEG (*P* ≤ 0.05  | Log(FC) | ≥1), with 1277 upregulated and 1733 downregulated genes. Therefore, the top 100 genes were selected for thermographic analysis (Figure 1(d), Supplementary Figure 1.2, Supplementary Table 3).

Finally, 62 common genes derived from the intersection of GEO-DEGs and TCGA-DEGs as potential DEGs were employed (Figure 1(b), Supplementary Table 4).

### 3.2. Candidate DEG WGCNA Analysis and Identification Key Modules

**Figure 2:**
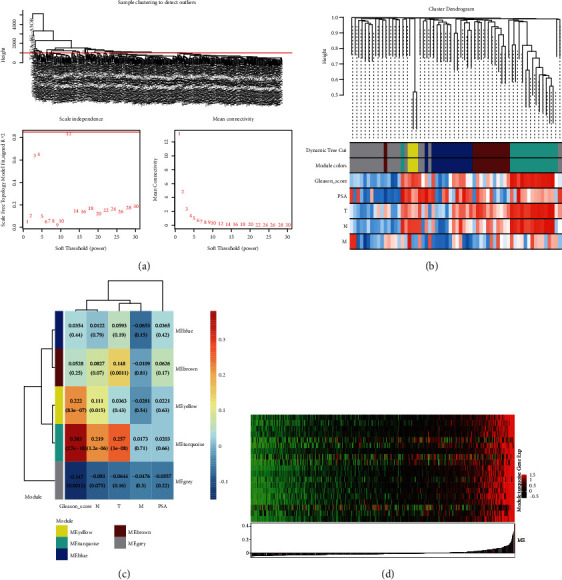
Construction of coexpression network and hub gene screening. (a) Analysis of the scale-free fit index (*R*^2^) and the mean connectivity with different soft-thresholding powers. At *R*^2^ > 0.8, the mean connectivity close to zero was considered an appropriate soft threshold. When we choose eight as our power, *R*^2^ = 0.85. (b) WGCNA module depicting the gene trait correlation plot showing the clustering dendrograms of genes. The clustering was based on 471 samples of TCGA-PRAD RNA-seq data. The color intensity varies positively with PSA, Gleason score, and T, N, and M stages. (c) The WGCNA module trait correlation plot. Overview of the modules generated by the WCGNA and their relationship with module eigengenes and the clinical traits of PCa. Each row represents a module, and each column represents a trait attribute. The blue color represents a negative correlation, and the red represents a positive correlation. (d) The gene expression in the green module.

**Figure 3:**
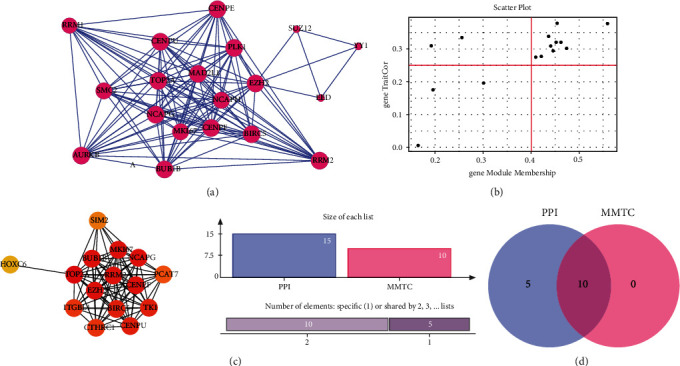
Construction of coexpression network and hub gene screening. (a) The protein-protein interaction network (PPI network) from each DEG module. (b) Scatterplot for ME magenta reveals the correlation between the module membership (MM) and the gene TraitCor (TC). The genes were selected from the green module by setting the membership degree of the green module to MM > 0.4 and TC > 0.25. (c) The first 12 hub genes are listed by using the Cytoscape software. (d) The generated 10 hub genes by combining MM/TC and PPI networks.

We performed WGCNA analysis on candidate DEGs to find the critical gene modules most relevant to the clinical characteristics of PCa based on the TCGA-PRAD dataset (Figures 2 and 3). Among them, the clinical characteristics of PCa matching the candidate DEGs mainly included the Gleason score, PSA, and TNM grades (Figure 2(b)). The soft parameter threshold was 12 (scale-free *R*^2^ = 0.85), the cluster height was 0.25, and the gene clustering method for similar expression profiles was the dynamic tree cut algorithm (Figure 2(a)). Finally, five modules were determined (Figures 2(b) and 2(c)); each branch within the hierarchical tree or the vertical line in the colored bar represents a gene. The genes not attributed to any module are gray. The module-feature correlation heatmap depicted that the green module correlated with clinical features. The Gleason score was the most significant (Person correlation value = 0.38, *P* = 2.66*E* − 18, Figure 2(c)). The gene expression in the green module developed a heatmap (Figure 2(d)), and there was a close relationship between these genes.

### 3.3. Key Modules MMTC and PPI Network to Screen Hub Genes

We selected 10 pivot genes from the green module by establishing the membership degree of the green module to MM > 0.4 and TC > 0.25 (Figure 3(b)). Then, we created a protein-protein interaction network (PPI network) for each module DEG based on the matching protein interaction network of the green module in Cytoscape software using the CytoHubba plug-in and the MCC screening approach [9] (Figure 3(a)). The first 12 hub genes are listed in Figure 3(c). Finally, 10 hub genes were generated by combining the MM/TC and PPI networks (EZH2, BUB1B, MK167, CENPF, NCAPG, TK1, CENPU, TOP2A, BIRC5, and RRM2; Figure 3(d)). We selected TOP2A, NCAPG, and BUB1B among the 10 hub genes listed above as the final important genes by combining MCC scores (Supplementary Table 5).

### 3.4. Internal and External Validation of Last Hub Gene Expression

Among the 10 central genes screened above, we selected 3 genes (TOP2A, NCAPG and BUB1B) as important central genes for the next step.

More significantly, three hub genes were validated using internal (TCGA) and external (GSE32571, GSE70770) validation datasets.

**Figure 4:**
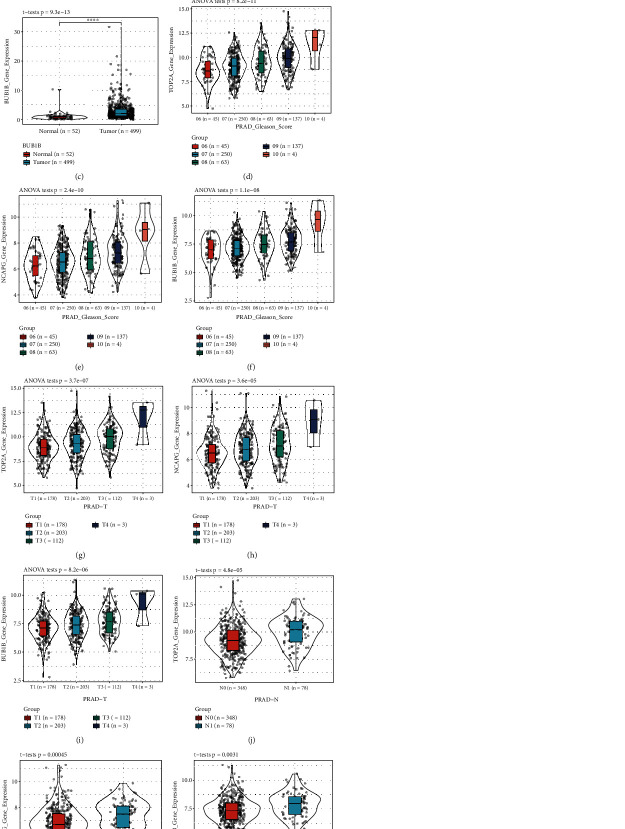
Validation of the gene expression levels of TOP2A, NCAPG, and BUB1B, by different datasets through various aspects. (a–c) TOP2A, NCAPG, and BUB1B gene expression levels between tumor and normal samples in the TCGA-PRAD dataset (internal validation dataset). (d–f) The correlation of TOP2A, NCAPG, and BUB1B with different Gleason scores (6, 7, 8, 9, and 10) in the TCGA-PRAD dataset (internal validation dataset). (g–i) The association between TOP2A, NCAPG, and BUB1B expressions and the different T stages (T2, T3, and T4) in the TCGA-PRAD dataset (internal validation dataset). (j–l) The expression of CCNA2, CKAP2L, NCAPG, and NUSAP1 in PCa samples with diverse T stages (N0, N1) in the TCGA-PRAD dataset (internal validation dataset). One-way analysis of variance (ANOVA) and Student's *t*-test was utilized to calculate statistical differences in these datasets.

Based on the clinical samples, the expression of these genes was significantly higher in the cancer group than in the matching normal control group, with *P* values of 3.6*E* − 07, 0.00021, and 9.3*E* − 13, respectively (Figures 4(a)–4(c)). In addition, TOP2A, NCAPG, and BUB1B were differentially expressed in different Gleason scores, T grades, and N grade PCa samples, and their expression levels were proportional to the sample Gleason score and T and N grades, *P* < 0.001 (Figures 4(d)–4(l)).

**Figure 5:**
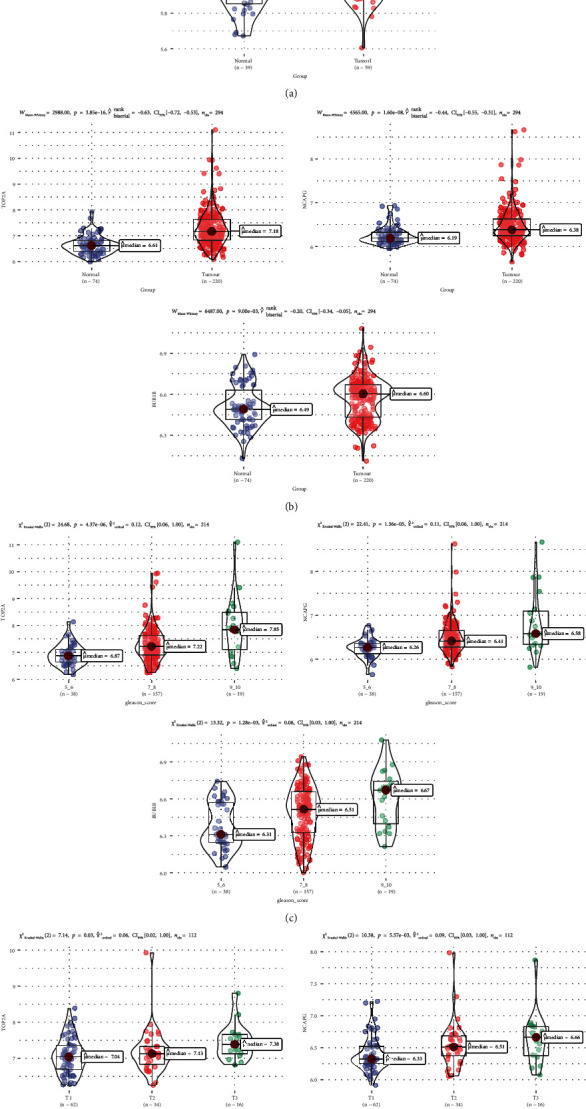
External validation of TOP2A, NCAPG, and BUB1B gene expression levels by different GEO datasets. (a) Gene expression levels of TOP2A, NCAPG, and BUB1B between PCa and normal samples in in the GSE32571 dataset. (b) TOP2A, NCAPG, and BUB1B gene expression differences between PCa and normal samples in the GSE70770 dataset. (c) Correlation of TOP2A, NCAPG and BUB1B with different Gleason scores (5, 6, 7, 8, 9, and 10) in the GSE70770 dataset. (d) Association between TOP2A, NCAPG, and BUB1B expressions and different T stages (T1, T2, and T3) in the GSE70770 dataset. Mann–Whitney and Kruskal-Wallis tests were utilized to calculate statistical differences in these datasets.

In addition, similar results were observed in external validation, the expression of 3 hub genes was significantly higher in the cancer group than in the matching normal control group (Figures 5(a) and 5(b)). The expression levels of 3 hub genes were proportional to the sample Gleason score and T grades (Figures 5(c) and 5(d)).

**Figure 6:**
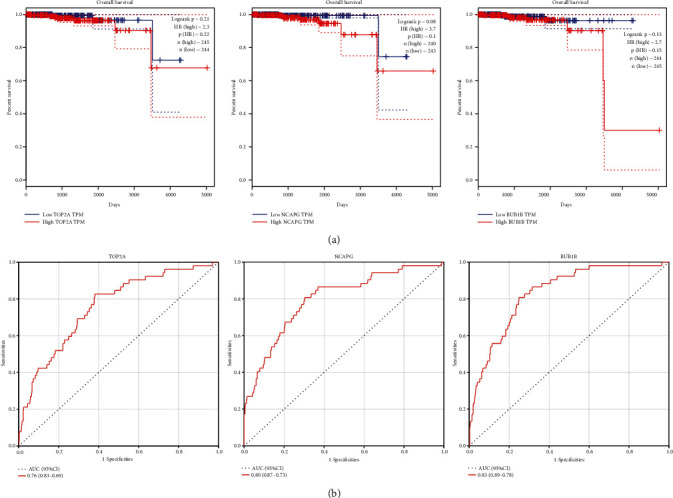
Validation of TOP2A, NCAPG, and BUB1B using survival analysis and ROC curve. (a) The correlation between TOP2A, NCAPG, and BUB1B overall survival time is based on the best separation in the TCGA-PRAD dataset (internal validation dataset). (b) Receiver operating characteristic (ROC) curves and area under the curve (AUC) statistics were undertaken to evaluate the ability of hub genes to distinguish PCa from normal samples with significant specificity and sensitivity in the TCGA dataset.

Furthermore, the KM and ROC curves based on the TCGA-PRAD dataset demonstrated that these genes were closely associated with clinical prognosis, having overall survival (Figure 6(a)) and TOP2A AUC = 0.76, NCAPG AUC = 0.80, and BUB1B AUC = 0.85 (Figure 6(b)), demonstrating their significant diagnostic and prognostic potentials as PCa biomarkers.

In addition, based on the UCAN online database, protein levels of these 3 genes were significantly higher in tumor tissues than in normal tissues and were positively correlated with Gleason score and T and N stages of PCa (Supplementary Figure 2).

### 3.5. Hub Gene GSVA and GSEA

**Figure 7:**
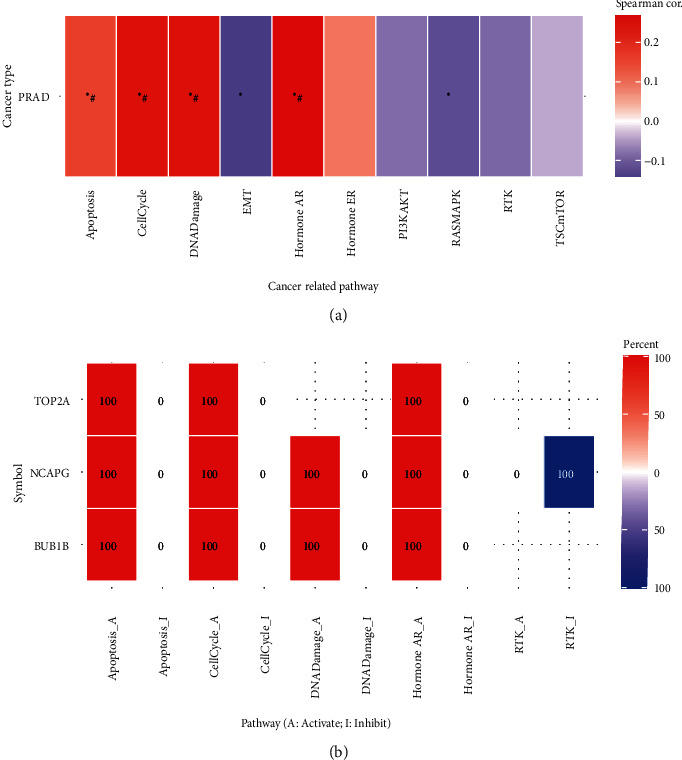
Gene set variation analysis (GSVA) of hub genes. (a) Heat plot summarizes the association between the GSVA score and the activity of cancer-related pathways in PCa. (b) Heatmap summarizes the percentage of PCa in the mRNA expression of hub genes (TOP2A, NCAPG, and BUB1B) that potentially affect pathway activity. The GSVA score represents the integrated level of gene set expression which is positively correlated with the gene expression. ^∗^*P* value ≤ 0.05; ^#^FDR ≤ 0.05.

GSVA and GSEA were utilized to investigate and evaluate the potential activities of the hub genes. According to the GSVA analysis, apoptosis, cell cycle, DNA damage, epithelial-mesenchymal transition (EMT), androgen receptor (hormone AR) pathways, among others, were associated with the hub genes in prostate cancer (Figure 7(a), Supplementary Table 6). Furthermore, the expression of these genes was positively correlated with the activation of the above pathways (Figure 7(b), Supplementary Table 7). Therefore, it was hypothesized that these hub genes could be linked to PCa and CRPC proliferation and medication resistance.

**Figure 8:**
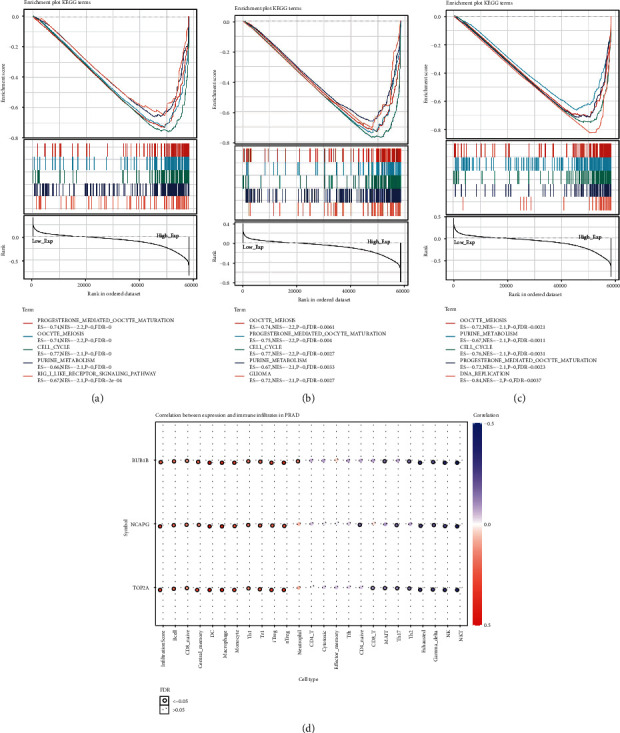
Gene set enrichment analysis (GSEA) and the relationship between hub genes expression and 24 immune cells. (a–c) The top five gene sets are enriched in the high-expression group of single hub genes according to the GSEA enrichment score. Each small bar represents a gene within the top five gene sets. It demonstrates the correlation between genes in the top five gene sets and the real hub genes. (d) The bubble plot summarizes the correlation between TOP2A, NCAPG, and BUB1B mRNA expression and 24 immune cell types infiltrate in PCa. Bubble size correlates with FDR significance. The black outline border indicates FDR ≤ 0.05.

Furthermore, the GSEA analysis of hub genes based on the TCGA data revealed that metabolic pathways, including “apoptosis” and “cell Cycle,” had higher enrichment scores in the high-expression group. Therefore, these genes were associated with the proliferation activation process and connection (Figures 8(a)–8(c), Supplementary Figure 3).

### 3.6. Hub Gene and Tumor Immune Infiltration Analysis

Previously, the relationship between hub gene expression and the metabolic pathways participating in prostate cancer was analyzed. Next, the association between hub gene expression and the relevant immune infiltrating cells in the sample was evaluated. The results showed that the expression of BUB1B, NCAPG, and TOP2A in the B cells, CD8_naive cells, monocytes, macrophages (macrophage), dendritic cells (DC), natural regulatory T cells (n T-regs), and the infiltration level induction of adaptive regulatory T cells (i T-regs) were positively correlated. However, it was negatively correlated with antitumor natural killer cancer cells (NK), NKT cells, Gamma_delta cells, exhausted cells, and CD4 T cells. The cell relationship was not apparent (Figure 8(d), Supplementary Figure 4).

### 3.7. Hub Gene Mutation

**Figure 9:**
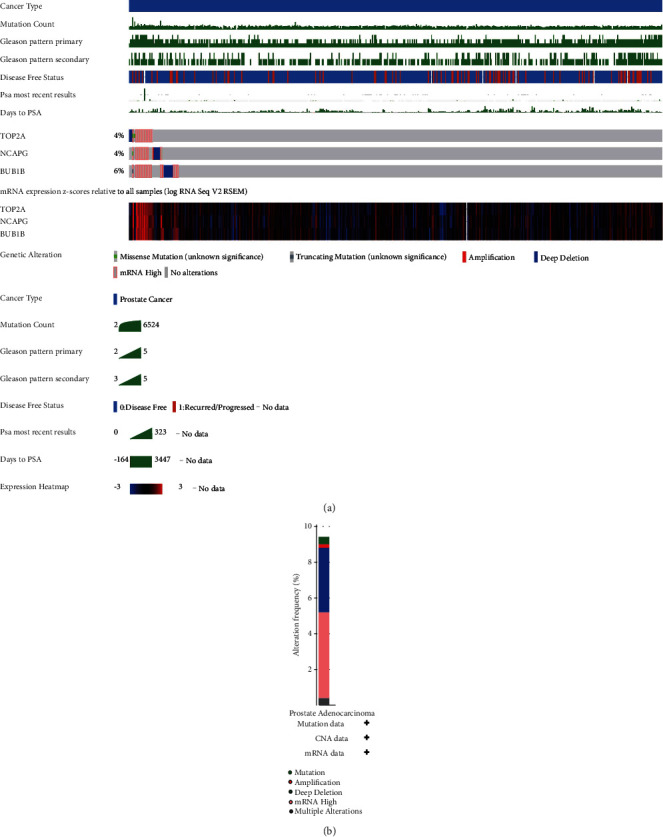
The mRNA expression patterns, genomic alterations, and methylation of the three hub genes. (a) The genetic alterations of the three hub genes based on the cBioPortal. The primary mutation types of the three hub genes were AMP, diploid, and deep deletion. BUB1B has the highest mutation rate (6%), while NCAPG and TOP2A have a 4% mutation rate. (b) Alteration frequency analysis of the three hub genes in TCGA-PRAD using the cBioPortal.

We analyzed the TCGA-PRAD mRNA expression data from the cBioPortal database and identified that the mutation types of the three hub genes were mostly AMP, diploid, and deep deletion. BUB1B had the highest mutation rate (6%), whereas NCAPG and TOP2A had a 4% mutation rate. Furthermore, 47 (9%) of all the three hub genes were mutated in 498 individuals (Figures 9(a) and 9(b)).

### 3.8. Hub Gene-Related Small-Molecule Drug Screening

**Figure 10:**
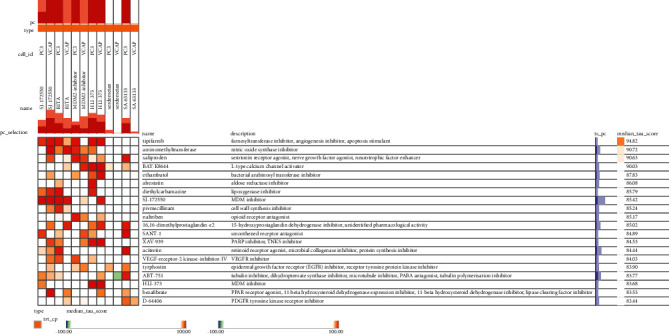
Small-molecule drugs were analyzed and screened in the green module DEGs. The small-molecule drugs, viz., tipifarnib, AMT, xaliproden and bay-K8644, were closely related to MDM inhibitors (median tau score > 90). Tipifarnib had the most significant median tau score = 94.82.

We utilized the CMap online database to assess DEGs in the green module to screen small-molecule medicines closely associated with PCa. There were 90 small-molecule medicines with a connection score (∣CS | >95) and an *n*‐sample ≥ 3 in the analysis results (Supplementary Table 8). Since all indicated a negative link, it was assumed that PCa could be slowed or stopped. A small-molecule medication (MDM inhibitor) was chosen for further investigation with a connection score of –99 and a target protein of MDM2/TP53. Using the PCL filtering approach and the PC3 and VCAP prostate cancer cell lines, the four small-molecule medications closely related to the MDM inhibitor (median tau score > 90) were finally obtained using the Touchstone software: farnesyltransferase, an angiogenesis inhibitor, and apoptosis; tipifarnib, an apoptosis promoter; aminomethyltransferase (AMT), a nitric oxide synthase inhibitor; xaliproden, a serotonin receptor agonist; and BAY-K8644, an L-type calcium channel activator. Tipifarnib had the highest median tau score of 94.82 among the comparable small-molecule medicines (Figure 10). These possible small-molecule medications can reverse PCa-induced gene expression and help develop targeted molecular therapies against PCa.

## 4. Discussion

The pathogenesis of prostate cancer is complicated, and metastases lead to medication resistance which is challenging to treat. As a result, proper identification is highly critical to therapy. In recent years, bioinformatics technology has provided many studies to screen biomarkers for malignant tumors [19–21]. However, only a few have made it into clinical practice. For the screening of 10 hub genes (EZH2, BUB1B, MK167, CENPF), NCAPG, TK1, CENPU, TOP2A, and BIRC5, RRM2), GEO and TCGA gene expression datasets were employed, as well as clinical information such as PSA, Gleason score, TNM staging, and more realistic screening approaches like WGCNA. The three genes, BUB1B, NCAPG, and TOP2A, have an excellent clinical diagnostic and predictive value. These genes are not only upregulated in prostate cancer tissues but also their expression levels are associated with the Gleason score, T and N staging, and overall survival analysis. The 5-year AUC values of the ROC curve were 0.6, 0.61, and 0.61, respectively. Furthermore, these genes could be linked to immune invading cells in prostate cancer and tumor therapy resistance. BUB1B (BUBR1), also known as the mitotic checkpoint for serine/threonine kinase B, belongs to the Bub1 family. A “destruction” box can degrade the targeted proteins during mitosis of the cell cycle [22]. BUB1B is abnormally expressed in cancers of the liver, pancreas, lung, breast, and other organs. Its clinical prognosis, especially its poor survival rate, is linked to the BUB1B gene expression [23–26]. Based on our findings, the expression of BUB1B in PCa tissue is significantly higher than in normal prostate tissue. Its expression is favorably associated with Gleason score and T and N staging and negatively correlated with the overall clinical survival based on the TCGA and GEO datasets. The findings of Zhong et al. [27] were also validated based on our findings. Our results revealed that BUB1B plays a critical role in the invasion and proliferation of PCa and is linked to various clinical outcomes.

The regulatory subunit, NCAPG, of the clusterin complex is essential for chromosomal condensation and stabilization in mitosis and meiosis. During mitosis, two threonine residues in the CAP-G subunit can be mutated, resulting in the CAP-G formation of the chromosome. Birth deformities and cancer have been associated with location defects [28]. The present study on NCAPG focuses on how it affects the cell cycle to enhance invasion, progression, and metastasis of liver cancer [29, 30]. Furthermore, NCAPG has been correlated with a poor clinical outcome in breast and lung cancer [30, 31]. Our results support the findings of Feng et al. and Arai et al. [32, 33], who observed that NCAPG expression is substantially associated with tumor stage and overall clinical survival rate.

Topoisomerase II (TOP2A) is a DNA topoisomerase II isoenzyme that regulates essential biological functions by modifying the topological structure of the chromosomal DNA [34]. Type II topological difference, anticancer, and antibacterial medicines are therapeutic targets for structural enzymes [35, 36]. TOP2A research has reached an advanced stage. The topoisomerase II inhibitor, etoposide phosphate (VP-16), has clear activity in individuals with metastatic castration-resistant prostate cancer (mCRPC), as evidenced by studies like Cattrini. Furthermore, TOP2A overexpression could be a biomarker for predicting mCRPC (excellent response to VP-16) [37]. Therefore, TOP2A has a higher diagnostic and prognostic value, as demonstrated in this study.

The tumor microenvironment (TME) includes tumors, stroma, and invading immune cells. Many studies have observed that tumor-infiltrating immune cells (TIIC) can modulate tumor prognosis, immunotherapy response rates, and chemotherapeutic efficacy [38, 39]. In prostate cancer progression, TIIC is also crucial. According to certain research, the PCa malignancy degree is directly associated with the infiltration trend of quiescent NK cells, memory B cells, M2 macrophages, and activated dendritic cells. The malignancy degree is adversely connected with naive B cells, active NK cells, and quiescent dendritic cells [40].

Furthermore, iNKT cells could slow PCa evolution by decreasing proangiogenic macrophages and boosting the regulatory mechanism of proinflammatory m1-like macrophages [41]. Moreover, a strong relationship was observed between the hub gene mRNA expression and immune cell infiltration in the study sample in this investigation. Therefore, TOP2A, NCAPG, and BUB1B are possible prognostic indicators associated with tumor-infiltrating immune cells in the tumor microenvironment. They could be evaluated as potential immunotherapy targets to enhance the clinical performance prognosis of PCa patients, especially CRPC patients.

The functional analysis of GSVA and GSEA revealed that the hub gene is primarily enriched in apoptosis, cell cycle, DNA damage, EMT, hormone AR, and other metabolic pathways. Therefore, they are linked to tumor growth and treatment resistance in the late stages of the tumor.

In this investigation, we found four related small-molecule medicines that could prevent PCa progression using the CMap database: tipifarnib, AMT, xaliproden, and BAY-K8644. Tipifarnib depicted the highest association among the three and was regulated by three hub genes. Tipifarnib is a farnesyltransferase inhibitor that is highly effective and selective and can treat various solid cancers. It treats HRA-mutated non-small-cell lung cancer [42] and pancreatic cancer [43] and is also being tested as a novel anticancer therapy for cervical squamous cell carcinoma [44].

Thus, tipifarnib could be beneficial in treating PCa, particularly CRPC; additional in vivo and in vitro testing is required.

However, there is no substantial relationship between the gene chosen and the clinical sample M staging and PSA levels. This lack of association could be due to the small sample size of M staging tumor patients (4 cases). Moreover, various aggravating factors and constraints could affect the PSA test value and the impact of sex in a person's life. The current work uses an open database for research and verification. In vivo and in vitro research are still required to ascertain the accuracy of these findings and better understand the specific roles and molecular mechanisms of the three identified biomarkers in the evolution of PCa.

## 5. Conclusion

We employed a combination of datasets, including GEO and TCGA and bioinformatics methods like WGCNA and cytoHubba, to screen for three hub genes (TOP2A, NCAPG, and BUB1B). Hub genes were confirmed and analyzed using GSVA, GSEA, cBioPortal, and CMap. Finally, TOP2A, NCAPG, and BUB1B could be exploited as potential PCa biomarkers. However, their reliability and specific mode of action are still under investigation.

## Data Availability

The study analyzed publicly available datasets that can be accessed here: https://www.cancer.gov/, The Cancer Genome Altas (TCGA), https://www.ncbi.nlm.nih.gov/, and Gene Expression Omnibus (GEO): GSE38241, GSE3325, and GSE46602.

## Supplementary Materials

Supplementary Materials Supplementary Table 1: three GEO datasets. The GSE38241, GSE3325, and GSE46602 microarray datasets were downloaded from the NCBI GEO dataset, which contained 67 tumor samples and 41 normal prostate samples. The table describes the dataset ID, references, and Gene Expression Omnibus Platform information of each GEO datasets. Supplementary Table 2: the 115 DEG_GEO. The NCBI Web analysis tool “GEO2R” was used to collect and evaluate the differences in the selected GEO datasets, and the 115 differential gene expression data matrix was generated with *P* ≤ 0.05 | Log FC | ≥1 as the standard. Adj.*P*.Val: adjusting *P* value. Log FC: logarithmic fold change. Supplementary Table 3: top 100 DEGs of the TCGA_PRAD data. DEGs of normal and cancer samples in TCGA-PRAD RNA-seq counts data was screened using the “EdgeR” R package based on the overdispersed Poisson model. Using *P* ≤ 0.05 | Log FC | ≥1 as criteria, the differential gene expression data matrix was obtained, and the top 100 target genes were selected for further analysis, 14 upregulated genes and 86 downregulated genes. Supplementary Table 4: the 62 common genes derived from the intersection of GEO-DEGs and the TCGA-DEGs. The 62 upregulated genes were obtained from overlapping parts of GEO-DEGs and TCGA-DEGs datasets for further analysis. Supplementary Table 5: top 10 in network node ranked by MCC method. Supplementary Table 6: the pathways were associated with the hub genes in prostate cancer. Supplementary Table 7: the expression of 3 hub genes was positively correlated with the activation of the pathways. Supplementary Table 8: the hub gene-drug-TOP 90 (∣Raw‐cs | >95, *N* ≥ 3). Ninety small-molecule drugs closely related to PCa were screened from DEG in the green module using CMap online database. Raw-SC ranged from -100 to +100; negative values indicated that the signal biology could be prevented or slowed down. Pert_id: Perturbagen ID; Raw-cs: raw-connection score; Cell iname: the type of cell line that the drug is treating; Moa: CMap mode of action analysis. Supplementary Table 9: information of abbreviations. The main abbreviations mentioned in the manuscript are shown in the table below. Supplementary Figure 1.1: heatmap of the top 115 DEG_GEO. Top 108 upregulated genes and top 7 downregulated genes were described by heatmap based on *P* value and Log FC value. Rows and columns represent genes and datasets, respectively. Red and blue indicate upregulation and downregulation of gene expression, respectively. Log FC: logarithmic fold change. Supplementary Figure 1.2: heatmap of the top 100 TCGA-PRAD counts data. Based on *P* value and Log FC, the heatmap showing the top 14 upregulated genes and the top 86 downregulated genes, respectively. Each row represents a gene, and each column represents a dataset. Red means upregulation, and blue means downregulation. Log FC was calculated according to “EdgeR” R package. Supplementary Figure 2: external validation of TOP2A, NCAPG, and BUB1B gene expression levels by UCAN online dataset. (A) Gene expression levels of TOP2A, NCAPG, and BUB1B between PCa and normal samples. (B) Correlation of TOP2A, NCAPG, and BUB1B with different Gleason scores (6, 7, 8, 9, and 10). (D) Association between TOP2A, NCAPG, and BUB1B expressions and different N stages (N0, N1). Student's *t*-test was utilized to calculate statistical differences in the UCAN online dataset. Supplementary Figure 3: gene set variation analysis (GSVA) of hub genes. (A) The detailed result of the GSVA score correlated with the specific pathway activity through the scatter plot, with a fitting line. (B–D) The boxplot depicts the pathway activity score between the high- and low-expression groups in TOP2A, NCAPG, and BUB1B. Supplementary Figure 4: hub gene and tumor immune infiltration analysis. (A) The detailed result of the mRNA expression of hub genes positively correlated with TOP 4 immune cell infiltrate through the scatter plot, with a fitting line. (B) The detailed result of the mRNA expression of hub genes negatively correlated with TOP4 immune cell infiltrate through the scatter plot, with a fitting line.
