# Supplementary material for: Identification of the Key Genes Involved in the Tumorigenesis and Prognosis of Prostate Cancer
Source: Comput Math Methods Med. 2022 Oct 5;2022:5500416. doi: 10.1155/2022/5500416 (PMC9556187; doi:10.1155/2022/5500416)

**Supplementary Materials**

**Supplementary Table1 Three GEO datasets**

The GSE38241, GSE3325, and GSE46602 microarray datasets were downloaded from the NCBI GEO dataset, which contained 67 tumor samples and 41 normal prostate samples. The table describes the Dataset ID, References and Gene Expression Omnibus Platform information of each GEO Datasets.

| Supplementary Table1 Three GEO datasets. | | | | |
| --- | --- | --- | --- | --- |
| Dataset | References | Platform | Tumor | Normal |
| GSE46602 | Mortensen | [HG-U133_Plus_2] Affymetrix Human Genome U133 Plus | 36 | 14 |
|  | et al^[1]^. | 2.0 Array |  |  |
| GSE3325 | Varambally | [HG-U133_Plus_2] Affymetrix Human Genome U133 Plus | 13 | 6 |
|  | et al^[2]^. | 2.0 Array |  |  |
| GSE38241 | Aryee et al^[3]^. | Agilent-014850 Whole Human Genome Microarray 4x44K | 18 | 21 |
|  |  | G4112F (Feature Number version) | | |

[1] Mortensen MM, Hoyer S, Lynnerup AS, et al. Expression profiling of prostate cancer tissue delineates genes associated with recurrence after prostatectomy [J]. Sci Rep, 2015, 5(16018).

[2] Varambally S, Yu J, Laxman B, et al. Integrative genomic and proteomic analysis of prostate cancer reveals signatures of metastatic progression [J]. Cancer Cell, 2005, 8(5): 393-406.

[3] Aryee MJ, Liu W, Engelmann JC, et al. DNA methylation alterations exhibit intraindividual stability and interindividual heterogeneity in prostate cancer metastases [J]. Sci Transl Med, 2013, 5(169): 169ra10.

**Supplementary Table 2 The 115 DEG_GEO**

The NCBI Web analysis tool "GEO2R" was used to collect and evaluate the differences in the selected GEO datasets, and the 115 differential gene expression data matrix was generated with *P*≤0.05∣LogFC∣≥1 as the standard.

Adj.*P*.Val, adjusting *P* Value

Log FC ,logarithmic fold change

| Supplementary Table 2 The 115 DEG_GEO | | | | | | |
| --- | --- | --- | --- | --- | --- | --- |
| Gene.symbol | logFC | adj.P.Val |  | Gene.symbol | logFC | adj.P.Val |
| AMACR | 3.80E+00 | 6.29E-14 |  | E2F3 | 5.91E-01 | 2.35E-03 |
| DLX1 | 3.13E+00 | 1.06E-18 |  | MYEF2 | 5.84E-01 | 2.62E-05 |
| BIRC5 | 2.91E+00 | 6.55E-11 |  | MS4A8 | 5.75E-01 | 4.46E-03 |
| HPN | 2.75E+00 | 2.29E-17 |  | PLEKHB1 | 5.62E-01 | 4.49E-03 |
| CENPF | 2.43E+00 | 1.16E-08 |  | ALDH3B2 | 5.62E-01 | 5.99E-04 |
| CGREF1 | 2.31E+00 | 1.07E-08 |  | CTHRC1 | 5.41E-01 | 6.23E-02 |
| EZH2 | 2.18E+00 | 7.00E-10 |  | EIF4EBP1 | 5.26E-01 | 9.04E-02 |
| NCAPG | 2.15E+00 | 1.94E-08 |  | MGAT4B | 5.17E-01 | 4.39E-09 |
| TK1 | 2.05E+00 | 1.07E-06 |  | BICD1 | 5.17E-01 | 3.02E-03 |
| CENPU | 2.04E+00 | 1.10E-07 |  | FNIP2 | 5.06E-01 | 4.34E-03 |
| PDLIM5 | 2.00E+00 | 6.84E-09 |  | ATP11A | 4.91E-01 | 4.39E-02 |
| CCNB1 | 1.72E+00 | 2.41E-08 |  | ABRACL | 4.80E-01 | 2.61E-02 |
| SMIM22 | 1.69E+00 | 3.84E-07 |  | ECT2 | 4.66E-01 | 4.76E-06 |
| ERG | 1.68E+00 | 2.62E-04 |  | SRM | 4.46E-01 | 5.74E-06 |
| TMC5 | 1.64E+00 | 3.57E-03 |  | LINC00665 | 4.44E-01 | 1.55E-04 |
| RAB39B | 1.57E+00 | 2.68E-05 |  | NIPA1 | 4.23E-01 | 4.46E-02 |
| DUS1L | 1.57E+00 | 1.44E-07 |  | MCF2L | 4.12E-01 | 4.74E-02 |
| PPP1R14B | 1.51E+00 | 4.44E-08 |  | CHD1L | 4.11E-01 | 2.39E-02 |
| EPCAM | 1.51E+00 | 8.06E-11 |  | TMTC4 | 4.06E-01 | 2.54E-02 |
| YPEL1 | 1.49E+00 | 1.45E-14 |  | APOF | 3.85E-01 | 3.71E-02 |
| ACSM1 | 1.42E+00 | 3.04E-03 |  | TDRD1 | 3.80E-01 | 9.43E-07 |
| RRM2 | 1.40E+00 | 1.34E-07 |  | TRAF7 | 3.74E-01 | 1.17E-03 |
| TOP2A | 1.33E+00 | 4.72E-06 |  | HECTD4 | 3.52E-01 | 1.29E-05 |
| AURKA | 1.32E+00 | 4.64E-09 |  | INSM1 | 3.12E-01 | 1.14E-02 |
| DNAH5 | 1.30E+00 | 2.40E-04 |  | C2orf72 | 3.11E-01 | 9.62E-04 |
| APOC1 | 1.28E+00 | 1.77E-04 |  | HPCAL1 | 3.07E-01 | 7.98E-03 |
| SOX4 | 1.27E+00 | 6.92E-07 |  | RAB40C | 2.98E-01 | 1.93E-05 |
| SLC29A2 | 1.27E+00 | 5.19E-10 |  | CYTH2 | 2.97E-01 | 4.12E-02 |
| SIM2 | 1.27E+00 | 1.26E-06 |  | FAM234B | 2.60E-01 | 9.51E-03 |
| PCAT7 | 1.27E+00 | 1.47E-10 |  | MKI67 | 2.14E-01 | 2.39E-03 |
| ARHGAP32 | 1.24E+00 | 3.12E-09 |  | LEF1-AS1 | 1.69E-01 | 2.29E-02 |
| RCC1 | 1.21E+00 | 3.19E-06 |  | ERVH-6 | 1.35E-01 | 7.91E-04 |
| GJB1 | 1.20E+00 | 3.50E-06 |  | SBK1 | 1.13E-01 | 7.53E-04 |
| NUSAP1 | 1.18E+00 | 2.00E-08 |  | HOXC6 | 1.05E-01 | 1.11E-02 |
| CHDH | 1.11E+00 | 1.34E-05 |  | CECR2 | 6.27E-02 | 8.01E-02 |
| CYP2J2 | 1.09E+00 | 8.85E-04 |  | XYLB | 5.47E-02 | 4.62E-02 |
| APOE | 1.07E+00 | 8.34E-05 |  | TUBB2A | -4.30E-01 | 3.78E-02 |
| TBX1 | 1.06E+00 | 1.69E-02 |  | KCNH8 | -4.52E-01 | 9.17E-03 |
| ANLN | 1.05E+00 | 2.63E-05 |  | ENC1 | -5.36E-01 | 2.86E-02 |
| MARCKSL1 | 1.04E+00 | 8.80E-12 |  | TRPM8 | -7.84E-01 | 3.69E-02 |
| PRR11 | 1.02E+00 | 1.92E-07 |  | OR51E2 | -1.32E+00 | 6.82E-02 |
| SLC19A1 | 1.00E+00 | 1.69E-09 |  | AGR2 | -2.18E+00 | 5.85E-05 |
| CRACR2B | 9.86E-01 | 1.48E-07 |  | SULF1 | -2.59E+00 | 3.64E-11 |
| CDK19 | 9.81E-01 | 6.11E-04 |  | ERGIC1 | 8.53E-01 | 3.56E-07 |
| ZIC2 | 9.65E-01 | 2.50E-06 |  | GART | 8.42E-01 | 1.07E-08 |
| CAMKK2 | 9.59E-01 | 1.08E-09 |  | MICALCL | 8.23E-01 | 1.36E-03 |
| MAL2 | 9.53E-01 | 1.78E-04 |  | PKIB | 8.07E-01 | 4.39E-02 |
| NETO2 | 9.37E-01 | 6.83E-04 |  | CRAT | 7.97E-01 | 4.24E-08 |
| ELL3 | 9.37E-01 | 2.29E-05 |  | UAP1 | 7.93E-01 | 6.74E-05 |
| FAM222A | 9.35E-01 | 6.73E-06 |  | AP1S3 | 7.92E-01 | 5.27E-04 |
| RAB17 | 9.18E-01 | 1.33E-10 |  | SPIN4 | 7.72E-01 | 1.60E-04 |
| COL1A1 | 8.98E-01 | 7.10E-05 |  | TRIB1 | 7.24E-01 | 8.13E-03 |
| IVD | 8.77E-01 | 3.61E-08 |  | CDC25A | 7.12E-01 | 4.21E-04 |
| VAV2 | 8.66E-01 | 9.43E-10 |  | NME1 | 7.09E-01 | 1.84E-05 |
| NUP210 | 6.36E-01 | 2.32E-04 |  | HIST1H3E | 6.91E-01 | 5.20E-08 |
| BUB1B | 6.36E-01 | 9.87E-08 |  | PCTP | 6.84E-01 | 3.95E-04 |
| ITGBL1 | 6.12E-01 | 1.44E-03 |  | KCNG3 | 6.64E-01 | 1.33E-04 |
| DLX2 | 6.07E-01 | 4.55E-04 |  |  |  |  |

**Supplementary Table 3 Top 100 DEGs of the TCGA_PRAD data**

DEGs of normal and cancer samples in TCGA-PRAD RNA-SEQ Counts data was screened using the "EdgeR" R package based on the over-dispersed Poisson model. Using *P*≤0.05∣LogFC∣≥1 as criteria, the differential gene expression data matrix was obtained, and the top 100 target genes were selected for further analysis, 14 up-regulated genes and 86 down-regulated genes.

| Supplementary Table 3 Top 100 DEGs of the TCGA_PRAD data | | | | | | |
| --- | --- | --- | --- | --- | --- | --- |
| Gene.symbol | logFC | PValue |  | Gene.symbol | logFC | PValue |
| MFSD2A | -5.986 | 1.31E-161 |  | PAEP | -9.267 | 7.08E-62 |
| SERPINA5 | -6.807 | 9.24E-157 |  | CRISP1 | -8.754 | 1.61E-61 |
| ACSL6 | -5.031 | 1.84E-149 |  | GSTM3 | -2.305 | 2.16E-60 |
| MCF2 | -5.295 | 8.07E-129 |  | EVA1A | -2.638 | 2.81E-60 |
| AKR1B1 | -3.896 | 1.30E-126 |  | FAM83A | -5.117 | 4.59E-60 |
| EMX2 | -6.814 | 1.81E-122 |  | FUT3 | -4.135 | 5.40E-60 |
| HOXB8 | -6.280 | 6.28E-120 |  | GCNT4 | -2.444 | 1.27E-59 |
| CLDN2 | -7.946 | 3.25E-118 |  | PRDM16 | -2.612 | 3.75E-59 |
| SPINK2 | -7.439 | 3.13E-112 |  | SLC13A2 | -6.332 | 6.21E-59 |
| KLHL14 | -4.277 | 1.75E-108 |  | SLCO4C1 | -4.845 | 7.86E-58 |
| NDRG4 | -3.375 | 5.67E-105 |  | SNAP25 | -3.086 | 8.17E-58 |
| SPINK13 | -5.587 | 7.34E-100 |  | SEMG2 | -9.530 | 9.40E-58 |
| PATE2 | -5.457 | 7.51E-96 |  | PAX2 | -4.107 | 1.39E-57 |
| RASL10B | -3.493 | 1.07E-95 |  | SLC31A2 | -2.143 | 6.28E-57 |
| WNT9B | -4.921 | 2.14E-92 |  | CLU | -2.598 | 2.95E-56 |
| CRTAC1 | -4.315 | 5.25E-92 |  | HOXC6 | 2.948 | 5.47E-55 |
| CYP19A1 | -4.616 | 2.32E-89 |  | UNC5B | -2.228 | 3.03E-54 |
| SLC2A9 | -2.722 | 4.20E-89 |  | SLC46A2 | -4.149 | 4.13E-54 |
| CYSLTR2 | -3.747 | 1.16E-85 |  | UGT2B7 | -5.644 | 4.72E-54 |
| ANO1 | -2.895 | 4.74E-82 |  | RAB11FIP2 | -1.285 | 8.55E-54 |
| TMEM114 | -9.079 | 1.41E-80 |  | HS3ST5 | -4.966 | 8.64E-54 |
| ANXA13 | -5.540 | 9.42E-80 |  | HOXB9 | -3.771 | 1.09E-53 |
| PIP | -7.364 | 6.45E-79 |  | LPL | -3.724 | 1.24E-53 |
| CA2 | -4.476 | 1.36E-78 |  | SIM2 | 2.229 | 6.71E-53 |
| KCP | -3.511 | 5.80E-78 |  | GNAO1 | -2.513 | 1.00E-52 |
| ABCG2 | -2.835 | 3.71E-77 |  | SEMG1 | -9.787 | 2.04E-52 |
| KCNJ5 | -3.139 | 1.18E-76 |  | PIK3C2G | -4.397 | 6.61E-52 |
| MGAM | -4.731 | 5.82E-76 |  | ZIC2 | 4.438 | 9.70E-52 |
| C3orf36 | -3.541 | 8.21E-76 |  | LPCAT2 | -1.852 | 6.15E-51 |
| PAQR8 | -2.593 | 1.38E-75 |  | KCNJ15 | -3.297 | 6.28E-51 |
| HOXB6 | -4.610 | 1.95E-75 |  | LRFN1 | 1.583 | 9.58E-51 |
| EPHA10 | 2.154 | 2.64E-73 |  | PLLP | -1.770 | 7.04E-50 |
| MRO | -3.124 | 7.93E-72 |  | AMACR | 3.247 | 7.05E-50 |
| NKX2-3 | 4.303 | 5.65E-70 |  | TMEM171 | -3.045 | 2.15E-49 |
| ATP13A4 | -4.287 | 1.15E-69 |  | RHOBTB2 | -1.266 | 3.18E-49 |
| WWC3 | -1.399 | 2.80E-69 |  | GPRC5B | -1.847 | 5.21E-49 |
| C1orf186 | -3.393 | 4.89E-69 |  | GLIS3 | -2.128 | 4.35E-48 |
| KIRREL3 | -3.042 | 2.06E-68 |  | KCNQ1 | -1.910 | 6.90E-48 |
| SLC16A12 | -3.789 | 8.92E-68 |  | ENSG | 1.773 | 9.99E-48 |
| PATE4 | -9.211 | 4.04E-67 |  | QPRT | -2.419 | 1.04E-47 |
| PTGES | -3.062 | 6.27E-67 |  | CGREF1 | 1.963 | 1.05E-47 |
| AQP2 | -9.323 | 6.46E-66 |  | PDK4 | -3.045 | 1.54E-47 |
| PLA2G4A | -2.337 | 2.51E-65 |  | G0S2 | -2.301 | 1.88E-47 |
| STAC2 | -4.442 | 3.05E-65 |  | SLC28A3 | -4.468 | 4.85E-47 |
| HPN | 2.491 | 1.19E-64 |  | ST6GALNAC4 | -1.815 | 1.67E-46 |
| PNMT | -4.681 | 5.80E-64 |  | ARHGEF40 | -1.469 | 2.23E-46 |
| ABHD6 | -1.368 | 1.04E-63 |  | PYCR1 | 1.360 | 2.41E-46 |
| SLC45A2 | 5.093 | 9.58E-63 |  | SLMAP | -1.155 | 2.72E-46 |
| FRMD3 | -2.260 | 1.55E-62 |  | ONECUT2 | 3.298 | 5.41E-46 |
| LAPTM4A | -1.004 | 1.77E-62 |  | ZIC5 | 5.077 | 1.04E-45 |

**Supplementary Table 4**

**The 62 common genes derived from the intersection of GEO-DEGs and the TCGA-DEGs**

The 62 up-regulated genes were obtained from overlapping parts of GEO-DEGs and TCGA-DEGs data sets for further analysis.

| Supplementary Table 4 The 62 common genes derived from the intersection of GEO-DEGs and the TCGA-DEGs | | | | | | |  |
| --- | --- | --- | --- | --- | --- | --- | --- |
|  |  |  |  |  |  |  |  |
| Target gene | logFC | PValue |  | Target gene | logFC | PValue |  |
| ACSM1 | 2.847 | 7.68E-31 |  | KCNG3 | 3.051 | 1.16E-30 |  |
| AMACR | 3.247 | 7.05E-50 |  | KCNH8 | 1.800 | 2.22E-13 |  |
| APOC1 | 2.294 | 8.22E-32 |  | MARCKSL1 | 1.382 | 1.39E-42 |  |
| APOE | 1.698 | 1.15E-21 |  | MICALCL | 1.209 | 5.62E-10 |  |
| APOF | 2.413 | 1.93E-29 |  | MKI67 | 1.515 | 2.36E-19 |  |
| BICD1 | 1.563 | 5.44E-27 |  | MS4A8 | 1.550 | 7.42E-11 |  |
| BIRC5 | 1.943 | 6.50E-25 |  | NCAPG | 1.738 | 8.59E-23 |  |
| BUB1B | 1.479 | 2.98E-23 |  | NETO2 | 2.064 | 1.49E-37 |  |
| C2orf72 | 2.331 | 6.07E-35 |  | NME1 | 1.027 | 5.56E-22 |  |
| CAMKK2 | 1.655 | 6.56E-36 |  | NUP210 | 1.224 | 2.87E-37 |  |
| CECR2 | 1.198 | 3.79E-18 |  | OR51E2 | 2.394 | 5.71E-24 |  |
| CENPF | 1.586 | 1.59E-18 |  | PDLIM5 | 1.590 | 2.43E-24 |  |
| CENPU | 1.040 | 7.94E-13 |  | PKIB | 1.646 | 1.48E-21 |  |
| CGREF1 | 1.963 | 1.05E-47 |  | PPP1R14B | 1.278 | 7.74E-26 |  |
| CRACR2B | 1.348 | 2.60E-19 |  | RAB17 | 1.380 | 2.38E-34 |  |
| CTHRC1 | 1.747 | 4.32E-17 |  | RRM2 | 2.012 | 7.66E-29 |  |
| CYP2J2 | 1.429 | 8.18E-16 |  | SBK1 | 1.415 | 1.53E-43 |  |
| DLX1 | 2.965 | 1.88E-28 |  | SIM2 | 2.229 | 6.71E-53 |  |
| DLX2 | 3.229 | 1.04E-34 |  | SLC19A1 | 1.431 | 1.38E-45 |  |
| DNAH5 | 2.293 | 2.35E-36 |  | SMIM22 | 1.358 | 0.000000013 |  |
| DUS1L | 1.071 | 1.02E-23 |  | TBX1 | 1.692 | 8.83E-19 |  |
| ELL3 | 1.301 | 9.04E-26 |  | TDRD1 | 2.962 | 3.73E-24 |  |
| EPCAM | 1.100 | 1.51E-26 |  | TK1 | 1.250 | 1.78E-17 |  |
| ERG | 2.471 | 2.68E-17 |  | TMC5 | 1.201 | 2.03E-15 |  |
| EZH2 | 1.457 | 4.52E-40 |  | TMTC4 | 1.033 | 6.01E-25 |  |
| FAM222A | 1.742 | 3.90E-25 |  | TOP2A | 1.366 | 6.60E-13 |  |
| GJB1 | 1.412 | 1.80E-20 |  | TRIB1 | 1.412 | 3.15E-19 |  |
| HOXC6 | 2.948 | 5.47E-55 |  | TRPM8 | 1.247 | 1.49E-11 |  |
| HPN | 2.491 | 1.19E-64 |  | TUBB2A | 1.367 | 1.69E-19 |  |
| INSM1 | 1.610 | 2.63E-10 |  | YPEL1 | 1.287 | 8.97E-41 |  |
| ITGBL1 | 1.573 | 9.36E-13 |  | ZIC2 | 4.438 | 9.70E-52 |  |

**Supplementary** **Table 5**

| Top 10 in network node ranked by MCC method |
| --- |
| \| Rank \| Name \| Score \|  \| Rank \| Name \| Score \| \| --- \| --- \| --- \| --- \| --- \| --- \| --- \| \| 1 \| BUB1B \| 5 \|  \| 4 \| BIRC5 \| 4 \| \| 1 \| NCAPG \| 5 \|  \| 4 \| EZH2 \| 4 \| \| 1 \| TOP2A \| 5 \|  \| 4 \| AURKA \| 4 \| \| 4 \| ANLN \| 4 \|  \| 4 \| NUSAP1 \| 4 \| \| 9 \| RRM2 \| 3 \|  \| 9 \| PRR11 \| 3 \| |

**Supplementary Table 6**

| The pathways were associated with the hub genes in prostate cancer |
| --- |
| \| Cancer type \| Pathway \| Estimate \| *P*_value \| FDR \| Method \| Statistic \| \| --- \| --- \| --- \| --- \| --- \| --- \| --- \| \| PRAD \| Apoptosis \| 0.160671748 \| 0.002499407 \| 0.01749585 \| Spearman's rank correlation rho \| 6101056.92 \| \| PRAD \| CellCycle \| 0.251582771 \| 1.74921E-06 \| 0.000015743 \| Spearman's rank correlation rho \| 5440226.874 \| \| PRAD \| DNADamage \| 0.250280524 \| 1.9858E-06 \| 0.000015886 \| Spearman's rank correlation rho \| 5449692.875 \| \| PRAD \| EMT \| -0.139511132 \| 0.008767737 \| 0.052606421 \| Spearman's rank correlation rho \| 8283079.07 \| \| PRAD \| Hormone AR \| 0.268555452 \| 3.13924E-07 \| 3.13924E-06 \| Spearman's rank correlation rho \| 5316852.866 \| \| PRAD \| Hormone ER \| 0.092963444 \| 0.08155772 \| 0.326230882 \| Spearman's rank correlation rho \| 6593226.954 \| \| PRAD \| PI3KAKT \| -0.081096022 \| 0.128866605 \| 0.326230882 \| Spearman's rank correlation rho \| 7858461.041 \| \| PRAD \| RASMAPK \| -0.115299467 \| 0.030561727 \| 0.152808637 \| Spearman's rank correlation rho \| 8107085.058 \| \| PRAD \| RTK \| -0.087128509 \| 0.102686626 \| 0.326230882 \| Spearman's rank correlation rho \| 7902311.044 \| \| PRAD \| TSCmTOR \| -0.036730761 \| 0.49213895 \| 0.49213895 \| Spearman's rank correlation rho \| 7535971.018 \| |

**Supplementary** **Table 7**

| The expression of 3 hub genes was positively correlated with the activation of the pathways |
| --- |
| \| Cancer type \| Symbol \| Pathway \| FDR \| Class \| Diff \| Entrez \| \| --- \| --- \| --- \| --- \| --- \| --- \| --- \| \| PRAD \| BUB1B \| Apoptosis \| 0.014849257 \| Activation \| 0.231482172 \| 701 \| \| PRAD \| BUB1B \| CellCycle \| 0.013047045 \| Activation \| 0.253055732 \| 701 \| \| PRAD \| BUB1B \| DNADamage \| 0.014849257 \| Activation \| 0.318887937 \| 701 \| \| PRAD \| BUB1B \| EMT \| 0.104142678 \| None \| -0.401241716 \| 701 \| \| PRAD \| BUB1B \| Hormone AR \| 0.001331484 \| Activation \| 0.480473084 \| 701 \| \| PRAD \| BUB1B \| Hormone ER \| 0.118515399 \| None \| 0.111590171 \| 701 \| \| PRAD \| BUB1B \| PI3KAKT \| 0.444824058 \| None \| -0.228296719 \| 701 \| \| PRAD \| BUB1B \| RASMAPK \| 0.444824058 \| None \| -0.192544708 \| 701 \| \| PRAD \| BUB1B \| RTK \| 0.839995547 \| None \| -0.044366846 \| 701 \| \| PRAD \| BUB1B \| TSCmTOR \| 0.85414126 \| None \| -0.034524663 \| 701 \| \| PRAD \| NCAPG \| Apoptosis \| 0.020468076 \| Activation \| 0.229650693 \| 64151 \| \| PRAD \| NCAPG \| CellCycle \| 0.006838634 \| Activation \| 0.284675223 \| 64151 \| \| PRAD \| NCAPG \| DNADamage \| 0.020468076 \| Activation \| 0.312873359 \| 64151 \| \| PRAD \| NCAPG \| EMT \| 0.860773999 \| None \| -0.036222838 \| 64151 \| \| PRAD \| NCAPG \| Hormone AR \| 0.043188753 \| Activation \| 0.28898271 \| 64151 \| \| PRAD \| NCAPG \| Hormone ER \| 0.084576649 \| None \| 0.121068988 \| 64151 \| \| PRAD \| NCAPG \| PI3KAKT \| 0.354402481 \| None \| -0.236745007 \| 64151 \| \| PRAD \| NCAPG \| RASMAPK \| 0.099702336 \| None \| -0.378276287 \| 64151 \| \| PRAD \| NCAPG \| RTK \| 0.043188753 \| Inhibition \| -0.330495769 \| 64151 \| \| PRAD \| NCAPG \| TSCmTOR \| 0.289791383 \| None \| -0.225188684 \| 64151 \| \| PRAD \| TOP2A \| Apoptosis \| 0.046276979 \| Activation \| 0.204401861 \| 7153 \| \| PRAD \| TOP2A \| CellCycle \| 0.034392018 \| Activation \| 0.22615511 \| 7153 \| \| PRAD \| TOP2A \| DNADamage \| 0.106203494 \| None \| 0.231732338 \| 7153 \| \| PRAD \| TOP2A \| EMT \| 0.610173219 \| None \| -0.123467385 \| 7153 \| \| PRAD \| TOP2A \| Hormone AR \| 0.034392018 \| Activation \| 0.366900604 \| 7153 \| \| PRAD \| TOP2A \| Hormone ER \| 0.12911915 \| None \| 0.113683435 \| 7153 \| \| PRAD \| TOP2A \| PI3KAKT \| 0.444667504 \| None \| -0.231182477 \| 7153 \| \| PRAD \| TOP2A \| RASMAPK \| 0.344705595 \| None \| -0.26331324 \| 7153 \| \| PRAD \| TOP2A \| RTK \| 0.444667504 \| None \| -0.131090579 \| 7153 \| \| PRAD \| TOP2A \| TSCmTOR \| 0.761829476 \| None \| -0.056790783 \| 7153 \| |

**Supplementary Table 8**

**The hubgene-drug-TOP 90 (∣Raw-cs∣>95,N≥3)**

Ninety small molecule drugs closely related to PCa were screened from DEG in the green module using CMap online database.Raw- SC ranged from -100 to +100, negative values indicated that the signal biology could be prevented or slowed down.

Pert_id ,Perturbagen ID; Raw-connection score, Raw-cs; Cell iname, the type of cell line that the drug is treating; Moa, CMap mode of action analysis.

| \| Supplementary Table 8 Hubgene-drug-TOP 90（∣Raw-cs∣>95,N≥3） \| \| \| \| \| \| --- \| --- \| --- \| --- \| --- \| \| Pert_id \| Cell_iname \| N sample \| Moa \| Raw_cs \| \| BRD-K33622447 \| A375 \| 3 \| CDK inhibitor \| -0.99 \| \| BRD-K62627508 \| A375 \| 3 \| MDM inhibitor \| -0.99 \| \| BRD-A34751532 \| MCF7 \| 5 \| HSP inducer \| -0.98 \| \| BRD-K37237504 \| A375 \| 3 \| JAK inhibitor \| -0.98 \| \| BRD-K73368362 \| THP1 \| 3 \| MEK inhibitor \| -0.98 \| \| BRD-A45889380 \| MCF10A \| 3 \| NFKB inhibitor\|Cytokine production inhibitor\|p53 activator \| -0.98 \| \| BRD-K92241597 \| HEPG2 \| 3 \| Peptidase inhibitor \| -0.98 \| \| BRD-K78431006 \| SKMEL5 \| 3 \| ALK inhibitor \| -0.97 \| \| BRD-M86331534 \| NKDBA \| 6 \| Androgen receptor antagonist\|AKT inhibitor \| -0.97 \| \| BRD-K34014345 \| MCF7 \| 5 \| Anti-inflammatory \| -0.97 \| \| BRD-K34014345 \| A549 \| 3 \| Anti-inflammatory \| -0.97 \| \| BRD-A50737080 \| A549 \| 2 \| ATM kinase inhibitor\|ATR kinase inhibitor \| -0.97 \| \| BRD-K68488863 \| HUVEC \| 2 \| Aurora kinase inhibitor\|FLT3 inhibitor\|VEGFR inhibitor \| -0.97 \| \| BRD-K70301465 \| HBL1 \| 3 \| BTK inhibitor \| -0.97 \| \| BRD-K52233191 \| HT29 \| 3 \| CDK inhibitor \| -0.97 \| \| BRD-K68336408 \| MCF10A \| 3 \| EGFR inhibitor \| -0.97 \| \| BRD-K33610132 \| HT29 \| 3 \| EGFR inhibitor \| -0.97 \| \| BRD-K21361524 \| A375 \| 3 \| Exportin antagonist \| -0.97 \| \| BRD-K63195589 \| MCF7 \| 5 \| Farnesyltransferase inhibitor \| -0.97 \| \| BRD-K03816923 \| HCC515 \| 5 \| MAP kinase inhibitor\|Protein kinase inhibitor \| -0.97 \| \| BRD-K73255294 \| A375 \| 3 \| MDM inhibitor \| -0.97 \| \| BRD-K64925568 \| A375 \| 3 \| MDM inhibitor \| -0.97 \| \| BRD-A45498368 \| HEPG2 \| 3 \| MTOR inhibitor \| -0.97 \| \| BRD-K25433859 \| A549 \| 3 \| Norepinephrine inhibitor\|Tricyclic antidepressant \| -0.97 \| \| BRD-K21680192 \| LNCAP \| 2 \| Topoisomerase inhibitor \| -0.97 \| \| BRD-K71223042 \| TMD8 \| 3 \| BTK inhibitor \| -0.96 \| \| BRD-K51313569 \| THP1 \| 2 \| CDK inhibitor \| -0.96 \| \| BRD-K51313569 \| LNCAP \| 2 \| CDK inhibitor \| -0.96 \| \| BRD-K10995081 \| MCF7 \| 5 \| Dopamine receptor antagonist \| -0.96 \| \| BRD-A03249105 \| MCF7 \| 4 \| Estrogen receptor antagonist \| -0.96 \| \| BRD-K21361524 \| MCF7 \| 3 \| Exportin antagonist \| -0.96 \| \| BRD-A48570745 \| YAPC \| 3 \| GABA receptor agonist \| -0.96 \| \| BRD-A68493689 \| PC3 \| 3 \| HCV inhibitor \| -0.96 \| \| BRD-K14618467 \| HBL1 \| 3 \| IKK inhibitor \| -0.96 \| \| BRD-K47983010 \| A375 \| 3 \| IKK inhibitor \| -0.96 \| \| BRD-K94841585 \| MCF7 \| 4 \| Laxative \| -0.96 \| \| BRD-K57080016 \| HCC515 \| 5 \| MEK inhibitor \| -0.96 \| \| BRD-K07955840 \| SKMEL5 \| 3 \| MTOR inhibitor\|PI3K inhibitor \| -0.96 \| \| BRD-A36331462 \| A549 \| 4 \| Proteasome inhibitor \| -0.96 \| \| BRD-K21025364 \| MDAMB231 \| 2 \| Protein tyrosine kinase inhibitor \| -0.96 \| \| BRD-K59469039 \| HCC515 \| 3 \| Tyrosine kinase inhibitor\|Angiogenesis inhibitor\|VEGFR inhibitor \| -0.96 \| \| BRD-K09907482 \| HCC515 \| 3 \| Tyrosine phosphatase inhibitor \| -0.96 \| \| BRD-K21718444 \| HEPG2 \| 2 \| Abl inhibitor\|Aurora kinase inhibitor\|FLT3 inhibitor \| -0.95 \| \| BRD-A43331270 \| HT29 \| 4 \| Adrenergic receptor antagonist\|Calcium channel blocker \| -0.95 \| \| BRD-K62609077 \| MCF7 \| 5 \| Adrenergic receptor antagonist \| -0.95 \| \| BRD-K68065987 \| SKMEL5 \| 3 \| AKT inhibitor \| -0.95 \| \| BRD-K24593301 \| PC3 \| 2 \| ALK inhibitor\|IGF-1 inhibitor \| -0.95 \| \| BRD-A50737080 \| CD34 \| 3 \| ATM kinase inhibitor\|ATR kinase inhibitor \| -0.95 \| \| BRD-K42436189 \| SHSY5Y \| 2 \| ATR kinase inhibitor \| -0.95 \| \| BRD-K75295174 \| A375 \| 3 \| Auora kinase inhibitor \| -0.95 \| \| BRD-K07881437 \| A375 \| 3 \| Aurora kinase inhibitor\|FGFR inhibitor \| -0.95 \| \| BRD-K14870255 \| HT29 \| 3 \| AXL kinase inhibitor \| -0.95 \| \| BRD-A35869383 \| MCF10A \| 3 \| BTK inhibitor \| -0.95 \| \| BRD-K31238592 \| MCF7 \| 4 \| CCK receptor antagonist \| -0.95 \| \| BRD-K51313569 \| JURKAT \| 2 \| CDK inhibitor \| -0.95 \| \| BRD-K51313569 \| VCAP \| 5 \| CDK inhibitor \| -0.95 \| \| BRD-K52233191 \| THP1 \| 2 \| CDK inhibitor \| -0.95 \| \| BRD-K51313569 \| YAPC \| 2 \| CDK inhibitor \| -0.95 \| \| BRD-K60997853 \| PC3 \| 2 \| CDK inhibitor\|Growth factor receptor inhibitor \| -0.95 \| \| BRD-K14109347 \| HEPG2 \| 2 \| CHK inhibitor \| -0.95 \| \| BRD-K89997465 \| MCF7 \| 5 \| Dopamine receptor antagonist \| -0.95 \| \| BRD-K46386702 \| MCF10A \| 3 \| EGFR inhibitor \| -0.95 \| \| BRD-K05151076 \| MCF7 \| 5 \| Estrogen receptor antagonist \| -0.95 \| \| BRD-K16730910 \| HCC515 \| 3 \| FGFR inhibitor\|VEGFR inhibitor \| -0.95 \| \| BRD-K42828737 \| HT29 \| 3 \| FLT3 inhibitor\|VEGFR inhibitor \| -0.95 \| \| BRD-K42828737 \| MCF7 \| 2 \| FLT3 inhibitor\|VEGFR inhibitor \| -0.95 \| \| BRD-K30466858 \| HCT116 \| 3 \| Glutathione transferase inhibitor \| -0.95 \| \| BRD-K78055238 \| YAPC \| 3 \| GSK inhibitor \| -0.95 \| \| BRD-K68548958 \| HUVEC \| 3 \| Histone acetyltransferase inhibitor \| -0.95 \| \| BRD-K81473043 \| MCF10A \| 2 \| HSP inhibitor \| -0.95 \| \| BRD-A13021932 \| A549 \| 4 \| ITK inhibitor \| -0.95 \| \| BRD-K12502280 \| SKMEL5 \| 3 \| JAK inhibitor\|FLT3 inhibitor \| -0.95 \| \| BRD-A12230535 \| HEPG2 \| 3 \| MDM inhibitor \| -0.95 \| \| BRD-K62627508 \| A549 \| 2 \| MDM inhibitor \| -0.95 \| \| BRD-K12343256 \| HCC515 \| 3 \| MEK inhibitor \| -0.95 \| \| BRD-K37687095 \| MCF10A \| 3 \| MEK inhibitor \| -0.95 \| \| BRD-K82244583 \| A375 \| 2 \| MEK inhibitor \| -0.95 \| \| BRD-K49865102 \| MCF7 \| 4 \| MEK inhibitor\|MAP kinase inhibitor\|Protein kinase inhibitor \| -0.95 \| \| BRD-K08316444 \| MCF7 \| 3 \| Mitochondrial inhibitor \| -0.95 \| \| BRD-K14821540 \| A375 \| 2 \| Mitochondrial oxidative phosphorylation uncoupler \| -0.95 \| \| BRD-A45889380 \| A549 \| 3 \| NFKB inhibitor\|Cytokine production inhibitor\|p53 activator \| -0.95 \| \| BRD-K88560311 \| MCF7 \| 5 \| PARP inhibitor \| -0.95 \| \| BRD-K96037667 \| VCAP \| 5 \| Progesterone receptor agonist \| -0.95 \| \| BRD-K16478699 \| A375 \| 3 \| RAF inhibitor \| -0.95 \| \| BRD-K56343971 \| HEPG2 \| 3 \| RAF inhibitor \| -0.95 \| \| BRD-K89464729 \| HCC515 \| 3 \| Rho associated kinase inhibitor \| -0.95 \| \| BRD-K99964838 \| HBL1 \| 3 \| Src inhibitor\|Abl inhibitor\|Bcr-Abl inhibitor \| -0.95 \| \| BRD-K37890730 \| HUVEC \| 2 \| Topoisomerase inhibitor \| -0.95 \| \| BRD-A31095847 \| HUVEC \| 3 \| Vasopressin receptor antagonist \| -0.95 \| \| BRD-K51816706 \| MCF7 \| 5 \| VEGFR inhibitor \| -0.95 \| |
| --- | --- | --- | --- | --- | --- | --- | --- | --- | --- | --- | --- | --- | --- | --- | --- | --- | --- | --- | --- | --- | --- | --- | --- | --- | --- | --- | --- | --- | --- | --- | --- | --- | --- | --- | --- | --- | --- | --- | --- | --- | --- | --- | --- | --- | --- | --- | --- | --- | --- | --- | --- | --- | --- | --- | --- | --- | --- | --- | --- | --- | --- | --- | --- | --- | --- | --- | --- | --- | --- | --- | --- | --- | --- | --- | --- | --- | --- | --- | --- | --- | --- | --- | --- | --- | --- | --- | --- | --- | --- | --- | --- | --- | --- | --- | --- | --- | --- | --- | --- | --- | --- | --- | --- | --- | --- | --- | --- | --- | --- | --- | --- | --- | --- | --- | --- | --- | --- | --- | --- | --- | --- | --- | --- | --- | --- | --- | --- | --- | --- | --- | --- | --- | --- | --- | --- | --- | --- | --- | --- | --- | --- | --- | --- | --- | --- | --- | --- | --- | --- | --- | --- | --- | --- | --- | --- | --- | --- | --- | --- | --- | --- | --- | --- | --- | --- | --- | --- | --- | --- | --- | --- | --- | --- | --- | --- | --- | --- | --- | --- | --- | --- | --- | --- | --- | --- | --- | --- | --- | --- | --- | --- | --- | --- | --- | --- | --- | --- | --- | --- | --- | --- | --- | --- | --- | --- | --- | --- | --- | --- | --- | --- | --- | --- | --- | --- | --- | --- | --- | --- | --- | --- | --- | --- | --- | --- | --- | --- | --- | --- | --- | --- | --- | --- | --- | --- | --- | --- | --- | --- | --- | --- | --- | --- | --- | --- | --- | --- | --- | --- | --- | --- | --- | --- | --- | --- | --- | --- | --- | --- | --- | --- | --- | --- | --- | --- | --- | --- | --- | --- | --- | --- | --- | --- | --- | --- | --- | --- | --- | --- | --- | --- | --- | --- | --- | --- | --- | --- | --- | --- | --- | --- | --- | --- | --- | --- | --- | --- | --- | --- | --- | --- | --- | --- | --- | --- | --- | --- | --- | --- | --- | --- | --- | --- | --- | --- | --- | --- | --- | --- | --- | --- | --- | --- | --- | --- | --- | --- | --- | --- | --- | --- | --- | --- | --- | --- | --- | --- | --- | --- | --- | --- | --- | --- | --- | --- | --- | --- | --- | --- | --- | --- | --- | --- | --- | --- | --- | --- | --- | --- | --- | --- | --- | --- | --- | --- | --- | --- | --- | --- | --- | --- | --- | --- | --- | --- | --- | --- | --- | --- | --- | --- | --- | --- | --- | --- | --- | --- | --- | --- | --- | --- | --- | --- | --- | --- | --- | --- | --- | --- | --- | --- | --- | --- | --- | --- | --- | --- | --- | --- | --- | --- | --- | --- | --- | --- | --- | --- | --- | --- | --- | --- | --- | --- | --- | --- | --- | --- | --- | --- | --- | --- | --- | --- | --- | --- | --- | --- | --- | --- | --- | --- | --- | --- | --- | --- | --- | --- | --- | --- | --- | --- | --- | --- | --- | --- | --- | --- | --- | --- | --- |

**Supplementary Table 9 Information of Abbreviations**

**The main abbreviations mentioned in the manuscript are shown in the table below.**

| Supplementary Table 9 Information of main abbreviations | |
| --- | --- |
| **Abbreviations** | **Full name** |
| PCa | Prostate cancer |
| CRPC | Castration-resistant prostate cancer |
| mCRPC | Metastatic Castration-Resistant Prostate Cancer |
| GEO | Gene Expression Omnibus |
| TCGA | The Cancer Genome Atlas |
| PRAD | Prostate Adenocarcinoma |
| DEG | Differentially Expressed Gene |
| WGCNA | Weighted correlation network analysis |
| PSA | Prostate-specific antigen |
| MCC | Maximal Clique Centrality |
| GSEA | Gene Set Enrichment Analysis |
| GSVA | Gene Set Variation Analysis |
| GSCA | Gene Set Cancer Analysis |
| CMAP | Connectivity map |
| PPI | Protein-Protein Interaction |
| TC | Trait Cor |
| MM | Module Membership |
| KM curve | Kaplan-Meier curve |
| ROC curve | The time-dependent receiver operating characteristic curve |
| AUC | Area Under the ROC Curve |
| MsigDB | The Molecular Signatures Database |
| PCL | Perturbagen Class |
| TNM | Tumor Node Metastasis |
| ME | Module eigengene |
| MDM | Murine double minute |
| ATM | Aminomethyl transferase |

**Supplementary Figure 1.1 Heatmap of the Top115 DEG_GEO**

TOP 108 up-regulated genes and TOP 7 down-regulated genes were described by Heatmap based on *P* value and Log FC value. Rows and columns represent genes and data sets, respectively. Red and blue indicate up-regulation and down-regulation of gene expression, respectively.

LogFC ,logarithmic fold change


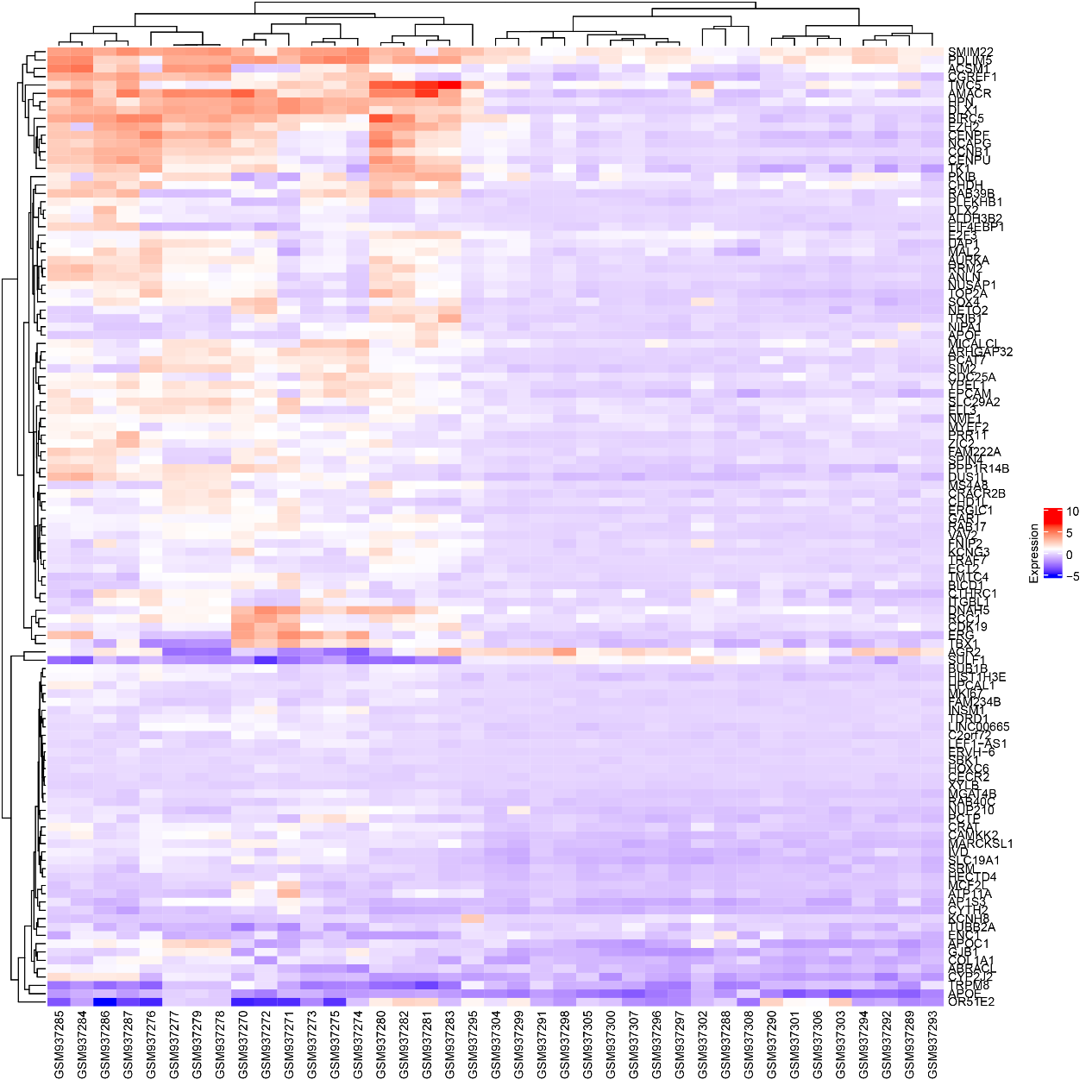


**Supplementary Figure 1.2**

**Heatmap of the top 100 TCGA-PRAD counts data.**

Based on *P* value and Log FC, the Heatmap showing the top 14 up-regulated genes and the top 86 down-regulated genes, respectively. Each row represents a gene, and each column represents a data set. Red means up-regulation, blue means down-regulation. Log FC was calculated according to "EdgeR" R package.


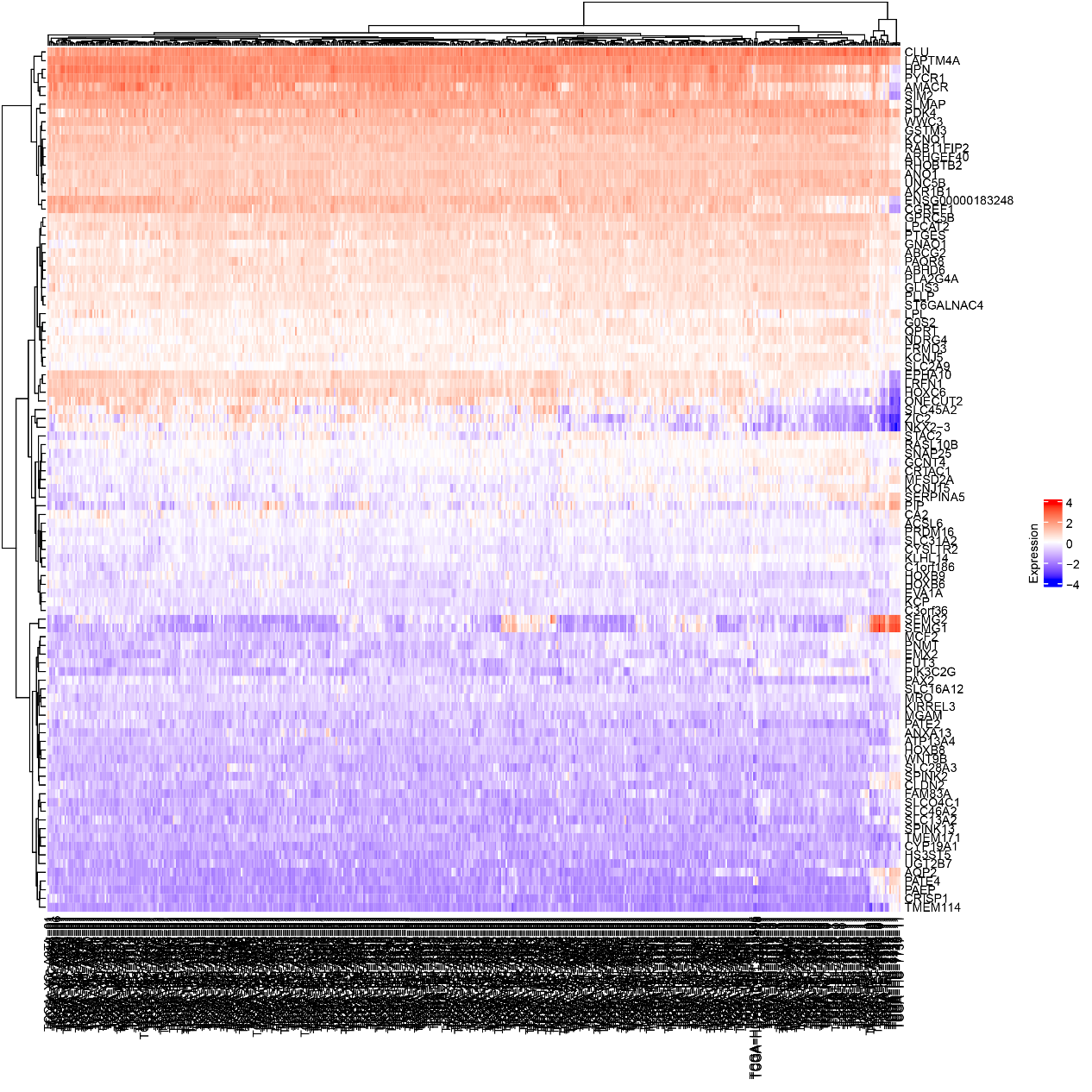


**Supplementary Figure 2**

**External validation of TOP2A, NCAPG and BUB1B gene expression levels by UCAN online dataset.**

(A) Gene expression levels of TOP2A, NCAPG and BUB1B between PCa and normal samples

(B) Correlation of TOP2A, NCAPG and BUB1B with different Gleason scores (6, 7, 8, 9, and 10)

(D) Association between TOP2A, NCAPG and BUB1B expression and different N stage (N0, N1).

Student's t-test was utilized to calculate statistical differences in UCAN online dataset.

| **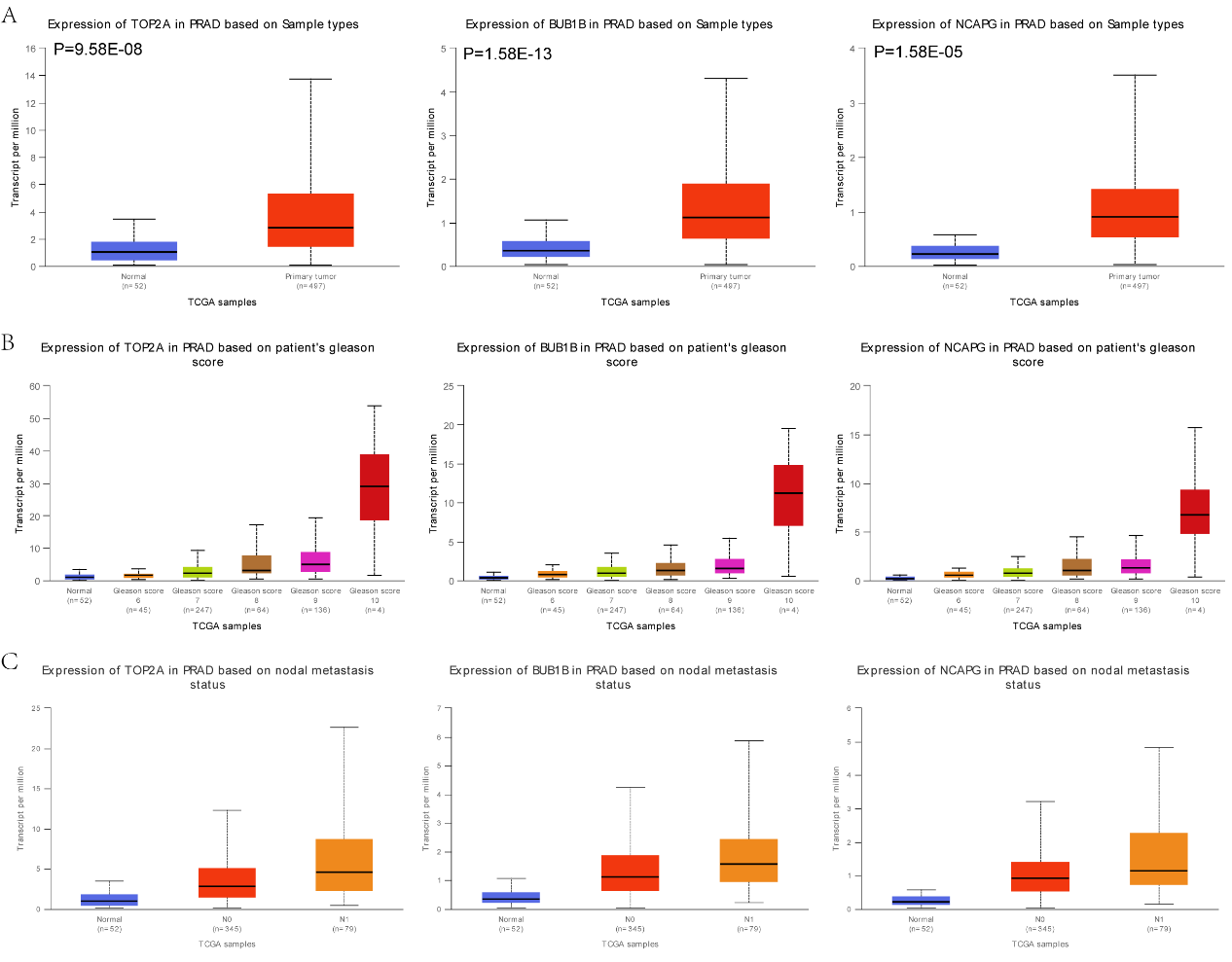** |
| --- |

**Supplementary Figure 3**

**Gene set variation analysis (GSVA) of hub genes**

(A)The detailed result of the GSVA score correlated with the specific pathway activity through the scatter plot, with a fitting line.

(B-D) The boxplot depicts the pathway activity score between the high and low expression groups in TOP2A, NCAPG, and BUB1B.


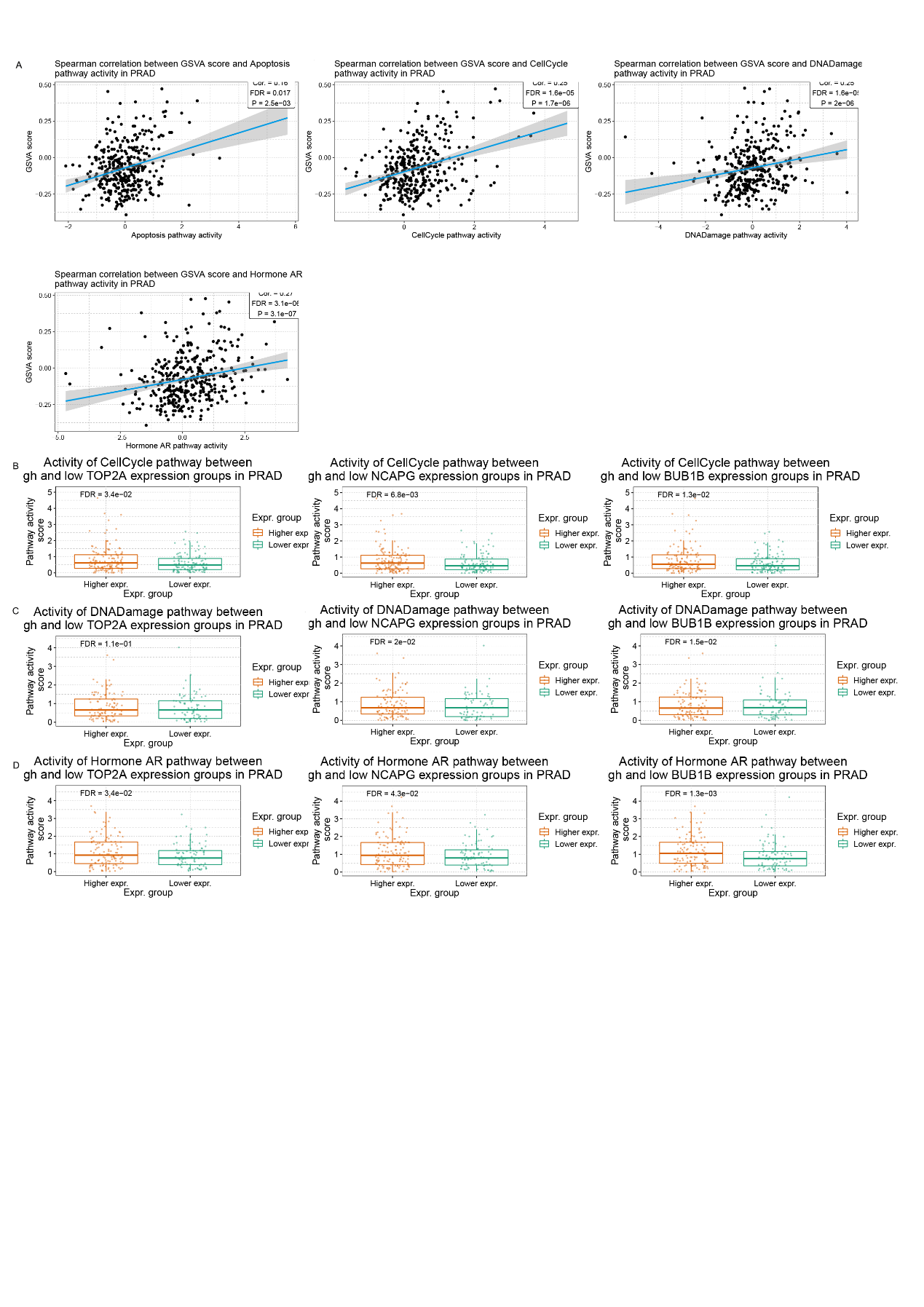


**Supplementary Figure 4**

**Hub gene and tumor immune infiltration analysis**

(A)The detailed result of the mRNA expression of hub genes positively correlated with TOP 4 immune cell infiltrate through the scatter plot, with a fitting line.

(B) The detailed result of the mRNA expression of hub genes negatively correlated with TOP4 immune cell infiltrate through the scatter plot, with a fitting line.


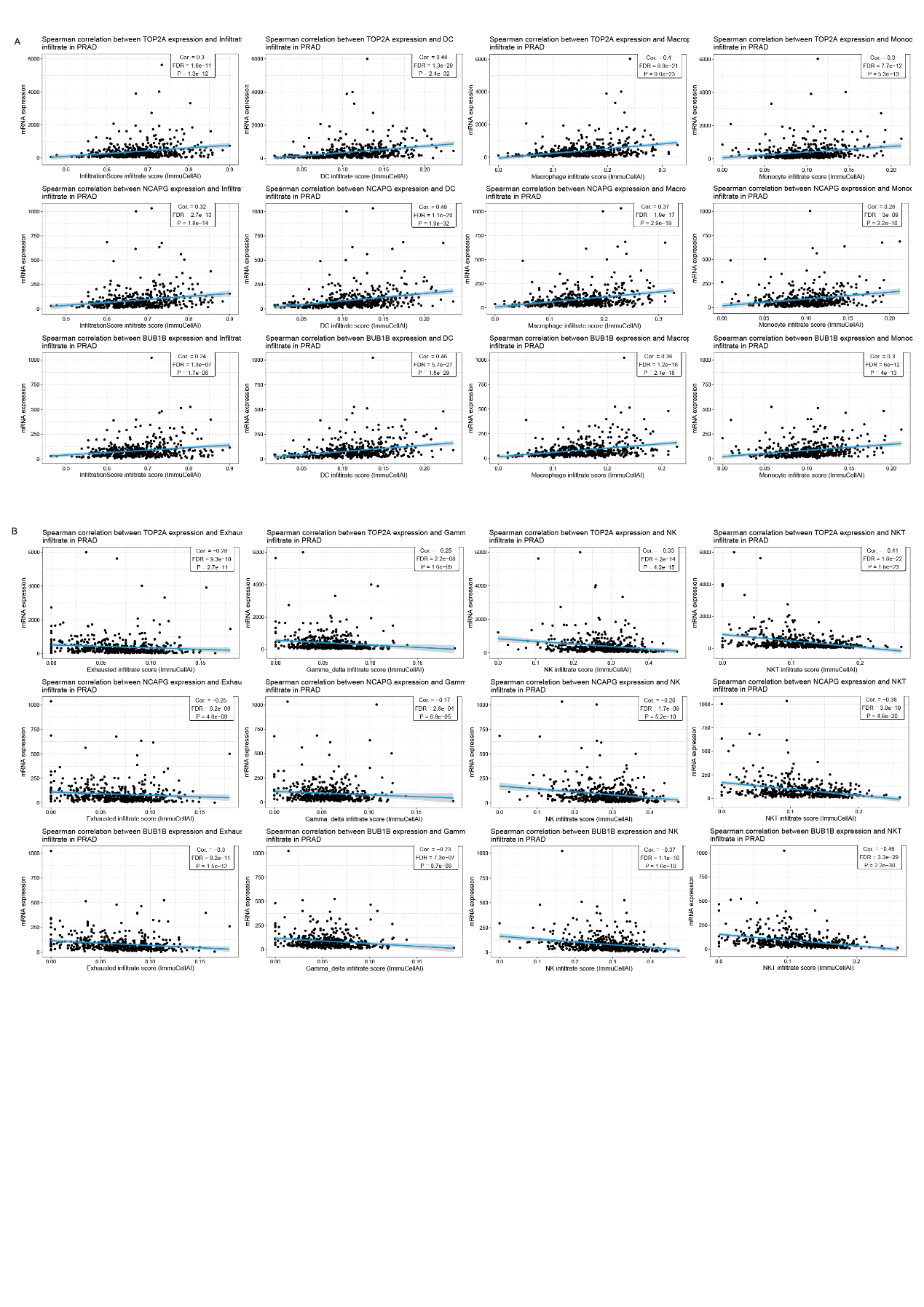

Supplement: Supplementary Materials — Supplementary Table 1: three GEO datasets. The GSE38241, GSE3325, and GSE46602 microarray datasets were downloaded from the NCBI GEO dataset, which contained 67 tumor samples and 41 normal prostate samples. The table describes the dataset ID, references, and Gene Expression Omnibus Platform information of each GEO datasets. Supplementary Table 2: the 115 DEG_GEO. The NCBI Web analysis tool “GEO2R” was used to collect and evaluate the differences in the selected GEO datasets, and the 115 differential gene expression data matrix was generated with P ≤ 0.05 | Log FC | ≥1 as the standard. Adj.P.Val: adjusting P value. Log FC: logarithmic fold change. Supplementary Table 3: top 100 DEGs of the TCGA_PRAD data. DEGs of normal and cancer samples in TCGA-PRAD RNA-seq counts data was screened using the “EdgeR” R package based on the overdispersed Poisson model. Using P ≤ 0.05 | Log FC | ≥1 as criteria, the differential gene expression data matrix was obtained, and the top 100 target genes were selected for further analysis, 14 upregulated genes and 86 downregulated genes. Supplementary Table 4: the 62 common genes derived from the intersection of GEO-DEGs and the TCGA-DEGs. The 62 upregulated genes were obtained from overlapping parts of GEO-DEGs and TCGA-DEGs datasets for further analysis. Supplementary Table 5: top 10 in network node ranked by MCC method. Supplementary Table 6: the pathways were associated with the hub genes in prostate cancer. Supplementary Table 7: the expression of 3 hub genes was positively correlated with the activation of the pathways. Supplementary Table 8: the hub gene-drug-TOP 90 (∣Raw‐cs | >95, N ≥ 3). Ninety small-molecule drugs closely related to PCa were screened from DEG in the green module using CMap online database. Raw-SC ranged from -100 to +100; negative values indicated that the signal biology could be prevented or slowed down. Pert_id: Perturbagen ID; Raw-cs: raw-connection score; Cell iname: the type of cell line that the drug is treat [file 5500416.f1.docx]
